# Modulating Surfactin Biosynthesis in *Bacillus subtilis* R31 Enhances Behavioural Traits and Biocontrol Efficacy Against Banana *Fusarium* Wilt

**DOI:** 10.1111/1751-7915.70261

**Published:** 2025-11-06

**Authors:** Hao‐Jun Chen, Yue Liu, Yun‐Shan Zhong, Ming‐Ze Li, Jia‐Jun Lai, Yan‐Yu Luo, Shao‐Li Huang, Shao‐Qing Liu, Guo‐Hui Yu, Yun‐Hao Sun, Ming‐Wei Shao

**Affiliations:** ^1^ College of Agriculture and Biology, Key Laboratory of Green Prevention and Control on Fruits and Vegetables in South China, Ministry of Agriculture and Rural Affairs, Zhongkai University of Agriculture and Engineering Guangzhou China; ^2^ Guangzhou Academy of Agricultural and Rural Sciences Guangzhou China

**Keywords:** *Bacillus subtilis* R31, biological control, *Fusarium* wilt of banana, surfactin

## Abstract

Surfactin, a lipopeptide antibiotic and quorum‐sensing (QS) mediator from *Bacillus subtilis* , has dual functions in microbial ecology and plant disease suppression. This study engineered *B. subtilis* R31 to overproduce *comK* and *phrC*, key regulators of surfactin biosynthesis, increasing surfactin yield by 45% compared to the WT strain. While elevated surfactin enhanced antimicrobial potential, *comK*‐mediated overproduction impaired biofilm formation and swarming motility, but rhizosphere colonisation was mostly unaffected. 16S rRNA sequencing of banana rhizospheres showed that surfactin selectively shaped the microbial community by enriching beneficial *Bacillus* species. Mechanistic studies confirmed surfactin's dual role as an antimicrobial and an intercellular signalling molecule for coordinated development in *Bacillus* populations. These results reveal the molecular mechanisms of R31‐mediated suppression of banana *Fusarium* wilt and offer a strategy for engineering synthetic microbial consortia by manipulating metabolic signalling pathways.

## Graphical abstract

This study demonstrates that the production of surfactin by *B. subtilis* R31 needs to be balanced. Excessive surfactin will weaken the colonisation ability and disease control effect of the strain. By targeting the regulation of the surfactin signalling pathway, synthetic microbial communities can be designed for sustainable disease control. This research provides a molecular basis for optimising biocontrol strains and emphasises the synergistic optimisation of metabolism and ecological adaptability.

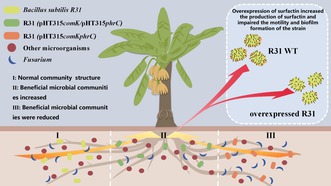

## 1 Introduction

First identified in *Bacillus subtilis* in 1968, surfactin is a cyclic lipopeptide known for its unique amphiphilic structure and wide range of biological activities (LozanoAndrade et al. 2025; Sonbhadra et al. 2025). This secondary metabolite is synthesised by nonribosomal peptide synthetases (NRPS) and features a *β*‐hydroxy fatty acid chain linked to a heptapeptide ring, with structural variations primarily occurring at the seventh amino acid residue (Cameotra and Makkar 2004; Marchant and Banat 2012). In addition to its well‐characterised antimicrobial activity, which operates through membrane disruption, surfactin demonstrates significant biotechnological potential due to its environmental compatibility, thermal stability and surfactant properties (Swaathy et al. 2014). These attributes have facilitated its adoption across agricultural, pharmaceutical and industrial sectors, aligning with global sustainable development initiatives.

The *srfAA‐AC* gene cluster is responsible for the orchestration of surfactin biosynthesis through a conserved NRPS mechanism, which involves the sequential activation of amino acids and the elongation of peptide chains (Chen et al. 2012; Xu et al. 2023) (Figure S1A). The investigation of *B. subtilis* R31 an endophytic strain isolated from the leaves of *Dendrobium orchids* showing efficacy against banana *Fusarium* wilt (Li et al. 2021) revealed a 27‐kb surfactin synthase cluster through anti‐SMASH prediction and liquid chromatography‐mass spectrometry (LC–MS) validation (Figure S1B). Plant pathogens rely on toxins, hydrolases and phytohormone perturbations to invade host tissues, and many bacteria coordinate these virulence factors through cell‐density‐dependent quorum sensing (QS) (Roca et al. 2024). Consequently, the ecological dominance of the strain under study stems not merely from its direct antimicrobial activity, but from surfactin's dual capacity to act as both an antibiotic that suppresses these QS‐controlled virulence determinants and as a QS signal that reshapes microbial interactions (Chen et al. 2020; Wen et al. 2021). Emerging evidence suggests that surfactin serves as a critical component in bacterial sociality. The role of surfactin is dual; namely, both by inducing controlled cell lysis, surfactin promotes the release of extracellular DNA (eDNA), which is essential for horizontal gene transfer while simultaneously providing immunity to the producer through mechanisms that remain to be elucidated (Rahman et al. 2021). Furthermore, the *comK* regulatory network integrates surfactin production with the development of competence; this master transcriptional activator regulates over 100 late competence genes through complex proteolytic control involving MecA/ClpCP complexes and quorum‐dependent ComS expression (Kovacs et al. 2013; Liu and Zuber 1998; Ogura et al. 2002). Importantly, the QS function of surfactin also extends to the modulation of carbon catabolite repression (CCR); *srfA* mutants are unable to utilise alternative carbon sources unless supplemented with surfactin or excess glucose, indicating a potential metabolic cross‐regulation (Chen et al. 2020).

The research result elucidates the evolutionary significance of surfactin for microbial kinship recognition. The proposed mechanism for kin discrimination allows *B. subtilis* to coordinate cooperative behaviours while simultaneously suppressing competitors, with surfactin functioning as both a molecular identifier and an ecological engineer (Henke and Bassler 2004; Patel et al. 2020). Kin recognition refers to the mechanism by which the same species or closely related species distinguish kin individuals (Kin) from non‐kin individuals (non‐Kin) through genetic or phenotypic signals, thereby regulating group behaviours (such as biofilm formation, collective movement) (Stefanic et al. 2015). The *Bacillus* species regulate the sharing and utilisation of surfactin through a kinship identification mechanism. This mechanism not only maintains the stability of cooperative behaviours among strains but also prevents nonrelated strains from engaging in free‐riding behaviour regarding shared resources (Drago et al. 2018; Liu et al. 2022). However, the natural yields of surfactin often pose limitations for functional studies (Kraigher et al. 2022), thereby necessitating genetic optimisation. The strategic hyperproduction of *comK* and *phrC* in strain R31 addresses this limitation while investigating two fundamental questions: (1) In what ways does surfactin‐mediated community assembly enhance the suppression of banana wilt? (2) Can the design of synthetic consortia utilise shared surfactin signalling for the sustainable control of phytopathogens?

## 2 Materials and Methods

### 2.1 Construction and Characterisation of R31 Surfactin Hyperproduction Strains

In this study, the plasmid vector pHT315‐*gfp* was employed as the backbone to construct surfactin hyperproduction constructs. The P43 constitutive promoter, the target gene with the Hix tag and the T7 transcriptional terminator were sequentially inserted into expression cassettes designed for the overproduction of *comK*, *phrC* and a dual *comK*/*phrC* gene combination. These recombinant plasmids were subsequently introduced into *B. subtilis* R31 using electroporation. The P43 promoter facilitated robust transcriptional activation of the target genes, while the T7 terminator enhanced transcriptional termination efficiency and mRNA stability. In the dual‐gene construct, an engineered ribosome binding site (RBS) was positioned between *comK* and *phrC*, enabling polycistronic expression. Upon transcription initiation by P43, the ribosome recognised the RBS to sequentially translate *comK* followed by *phrC* from the same mRNA strand. Successful plasmid integration and expression in R31 were confirmed through colony PCR and Western blot analysis, validating the establishment of the hyperproduction strains.

To facilitate expression verification, His‐tags were incorporated during the gene amplification process. The P43 promoter was amplified from the *B. subtilis* R31 genome, while the His‐tag and T7 transcription terminator were sourced from the pET28a (+) vector. These DNA fragments were fused using overlap extension (fusion) PCR, yielding amplified fragments of 1101 bp (*comK*), 645 bp (*phrC*) and 1264 bp (*comK*/*phrC* dual cassette). The pHT315‐*gfp* expression vector was enzymatically digested with EcoRI, and the fusion products were inserted using seamless cloning techniques to construct three hyperproduction plasmids: pHT315‐*comK*, pHT315‐*phrC* and pHT315‐*comKphrC*. These recombinant vectors were transformed into *Escherichia coli* DH5α for propagation, and PCR analysis confirmed insertion sizes of 1349 bp, 893 bp and 1512 bp respectively. Verified plasmids were then electroporated into *B. subtilis* R31. Erythromycin‐resistant transformants were selected on LB agar and confirmed by colony PCR, followed by sequencing for insert validation. Gene expression was assessed by Western blotting to detect His‐tagged proteins and quantitative real‐time PCR (qRT‐PCR) to determine relative transcript abundance using the 2^−ΔΔCT^ method. All data were statistically analysed and visualised using GraphPad Prism 9.0.2.

### 2.2 Biological Functions Validation of R31 Surfactin Hyperproduction Strains

To evaluate the functional consequences of enhanced surfactin synthesis, *B. subtilis* R31 strains were engineered to overexpress *comK*, *phrC* or *comKphrC*. While these constructs successfully increased surfactin production, the broader effects on bacterial physiology and ecological traits required further investigation. Specifically, the effect of regulatory gene overexpression on key phenotypes (e.g., surfactin yield, biofilm formation, swarming motility and root colonisation efficiency in banana plants) was investigated. To assess these parameters, this study conducted a comparative analysis among five strains: WT R31, vector‐only control R31 (pHT315) and the three recombinant strains (e.g., R31) (pHT315‐*comK*), R31 (pHT315‐*phrC*) and R31 (pHT315‐*comKphrC*). Initial phenotypic assays provided insight into the effects of surfactin hyperproduction on bacterial behaviour and environmental fitness. Additionally, transcriptomic profiling was performed, and DEGs were analysed using KEGG pathway enrichment. This approach enabled investigation of global transcriptional reprogramming associated with *comK* and *phrC* overexpression, as well as identification of specific metabolic and signalling pathways contributing to the observed phenotypic adaptations. All strains were activated by streaking onto LB agar plates and incubated at 37°C for 12 h. Individual colonies were picked and inoculated into 5 mL of LB broth for overnight shaking at 37°C, generating secondary seed cultures. A 1% aliquot of this culture was then transferred into 20 mL of fresh LB broth and incubated at 37°C for 4 h to yield the tertiary seed culture. For fermentation, 1% of the tertiary culture was inoculated into 200 mL of nutrient broth (NB) and incubated with shaking at 37°C for 48 h.

### 2.3 R31 Surfactin Extraction of Crude Products

Crude surfactin was extracted from the NB fermentation broth of each *B. subtilis* R31 strain through acid precipitation. Following fermentation, cultures were transferred to 50‐mL centrifuge tubes and centrifuged at 10000 rpm for 10 min at 4°C to remove bacterial cells. The resulting supernatant was adjusted to pH 2.0 with 1 N HCl and stored at 4°C overnight to facilitate surfactin precipitation. After overnight incubation, the precipitate was collected by centrifugation under the same conditions and further purified by triple extraction with spectroscopy‐grade methanol. The methanol extract was concentrated using a rotary evaporator, and the resulting residue was redissolved in 100 μL of chromatography‐grade methanol, filtered through a 0.22 μm membrane and stored at 4°C for subsequent analysis. For quantification, a standard surfactin solution was prepared by dissolving 10 mg of standard surfactin powder in 1 mL of chromatography‐grade methanol to achieve a final concentration of 10 mg/mL. HPLC was performed using an Agilent 1260 Infinity III system equipped with a CNW Athena C18‐WP column (4.6 × 250 mm). The mobile phase consisted of solvent A (0.1% trifluoroacetic acid in water) and solvent B (0.1% trifluoroacetic acid in chromatography‐grade acetonitrile, Maclean Biological Reagents, CAS: 75–05‐8). The column temperature was maintained at 35°C, with a flow rate of 0.85 mL/min, detection wavelength at 210 nm and injection volume set to 20 μL. The elution program was as follows: 0–9 min, 40:60 (A:B); 9.01–20 min, 7:93 (A:B) and 20.1–25 min, 95:5 (A:B). Each sample (e.g., R31 WT, R31 (pHT315), R31 (pHT315‐*comK*), R31 (pHT315‐*phrC*) and R31 (pHT315‐*comKphrC*)) was analysed in triplicate under identical conditions. The concentration of surfactant in each sample was calculated using the HPLC—external standard method formula:

$$\text{Surfactin}\;\left(\mathrm{mg}/L\right)=\frac{A1}{A2}\times C\times D$$

A1: Processing the main peak area of the sample.

A2: Area of the main peak of the standard sample.

C: Standard substance concentration (mg/L).

D: Total dilution factor of the fermentation broth.

### 2.4 Crude Extract Haemolytic Activity of Overexpressed Strain Surfactin

Haemolytic activity of the crude surfactin extracts was assessed using sheep blood agar plates. The blood agar medium was prepared by combining 10‐g casein trypsin digest, 3 g heart‐trypsin digest, 1 g corn starch, 5 g meat peptone, 5 g yeast extract, 5 g NaCl and 15 g agar powder in distilled water to a final volume of 1 L, adjusting the pH to 7.3. After sterilisation, the medium was cooled to 50°C, and 50–100 mL of sterile, defibrinated sheep blood was added. The mixture was gently agitated, poured into sterile Petri dishes and stored at 4°C in the dark until use. Prior to the assay, blood agar plates were equilibrated to room temperature in a 37°C incubator. Subsequently, 10 μL of surfactin crude extract from WT R31, R31 (pHT315‐*comK*), R31 (pHT315‐*phrC*), R31 (pHT315‐*comKphrC*), along with chromatographic‐grade methanol (solvent control) and a 10 mg/mL surfactin standard solution (positive control), were spotted onto the surface of each plate. Plates were air‐dried in a laminar flow hood and incubated at 37°C for 24 h.

### 2.5 Growth Curve Determination of R31 Hyperproduction Strains of *B. subtilis*

To assess the growth characteristics of *B. subtilis* R31 and its surfactin hyperproduction strains, glycerol stocks of each strain were streaked onto LB agar plates and incubated at 37°C for 12–24 h for activation. Single colonies were then inoculated into 5 mL of LB broth and cultured at 37°C with shaking at 180 rpm for 12 h to obtain secondary seed cultures. For strains harbouring plasmid constructs, erythromycin was added to a final concentration of 50 μg/mL; this antibiotic selection was consistently maintained in all subsequent cultures. The secondary cultures were then subcultured into fresh LB broth (with or without erythromycin, as appropriate) and incubated under identical conditions to reach log phase. Optical density (OD) at 600 nm (OD_600_) was measured every hour from 1 h to 10 h using a spectrophotometer to monitor bacterial growth dynamics. Growth curves were generated by plotting OD_600_ against time, and data were analysed using GraphPad Prism 9.0.2 to evaluate differences in growth rate and population density among the strains.

### 2.6 Differential Determination of Ability to Undergo Biofilm Formation in *B. subtilis* R31 Surfactin Hyperproduction Strain

The biofilm‐forming capacity of *B. subtilis* R31 and its surfactin hyperproduction strains was evaluated on both solid and liquid MSgg media. For solid surface assays, tertiary seed cultures prepared as previously described were normalised to equal concentrations, and 10 μL of each strain was spotted onto MSgg agar plates. After drying, plates were incubated at 37°C for approximately 72 h. Colony morphology was then visually assessed and documented to evaluate biofilm structure and architecture. For liquid surface assays, MSgg broth and the respective tertiary seed cultures were dispensed into 12‐well plates with four biological replicates per strain. Biofilm development was monitored at 24 h and 48 h. At each time point, planktonic cells were removed without disturbing the surface‐associated biofilm. Residual liquid was blotted with sterile filter paper, and wells were stained with 1% crystal violet solution for 20 min. Excess stain was gently rinsed off with sterile double‐distilled water until the wash remained clear, indicating complete removal of unbound dye. The adhered biofilm was then solubilised with 33% glacial acetic acid, and the OD at 570 nm (OD_570_) was measured using a microplate reader to quantify the biofilm biomass.

### 2.7 Swimming Capacity Determination of Hyperproduction *B. subtilis* R31 Strains

To evaluate motility characteristics, five *B. subtilis* R31 strains (e.g., WT R31, R31 (pHT315), R31 (pHT315‐*comK*), R31 (pHT315‐*phrC*) and R31 (pHT315‐*comKphrC*)) were streaked onto LB agar plates and incubated at 37°C for activation. Single colonies were then inoculated into 5 mL of LB broth and cultured at 37°C with shaking for 12 h to generate secondary seed cultures. For plasmid‐containing strains, erythromycin was added to a final concentration of 50 μg/mL, a condition maintained throughout all subsequent culturing steps. The secondary cultures were then transferred into fresh 5‐mL LB broth and incubated for an additional 4 h to obtain tertiary seed cultures, which were subsequently diluted to OD_600_ to ensure uniform inoculum density. Swarming motility was assessed on NA plates containing 0.5% agar. Fresh medium was prepared and poured into Petri dishes, which were left in a sterile laminar flow hood to dry for 30 min, followed by a 10 min air drying period. A 2 μL aliquot of the tertiary seed culture was spotted at the centre of each plate, and plates were incubated statically at 37°C. The extent of colony expansion was monitored to evaluate group motility. Sliding motility was assessed similarly using 0.7% LB semisolid agar plates. Plates were prepared, dried under sterile conditions and inoculated with 2 μL of the same tertiary seed culture. Plates were incubated under identical conditions, and colony diffusion was measured to determine the extent of sliding ability.

### 2.8 Colonisation Ability Determination of a Strain Hyperproduction *B. subtilis* R31 in Banana Roots

Preparation of *B. subtilis* R31 fermentation cultures was performed following the same methodology described previously. From the final fermentation broth, 0.5 mL of bacterial suspension was collected, with a colony‐forming unit (CFU) count estimated at approximately 1 × 10^9^ CFU/mL. The suspension was centrifuged at 6000 rpm for 10 min at 4°C to pellet the cells. The resulting supernatant was discarded, and the pellet was resuspended in sterile distilled water. This washing step was repeated once more to ensure the removal of residual media components. The bacterial pellet was then resuspended and diluted in sterile water to a final working concentration of 1 × 10^8^ CFU/mL, which was used as the inoculum for subsequent experiments or stored on ice as a backup.

The colonisation ability of *B. subtilis* R31 surfactin hyperproduction strains in banana roots was assessed using a hydroponic system. Tissue‐cultured Brazilian banana (*Musa* spp.) seedlings were initially transplanted into sand‐filled basins to promote root development. Once adequate root growth was observed, seven uniform seedlings were selected. The roots were gently rinsed with sterile water to remove residual substrate and transferred into sterile glass culture jars (jam bottles) for hydroponic cultivation. Each seedling was supplied with Hoagland nutrient solution and maintained under controlled conditions for 1 week to stabilise prior to bacterial inoculation. After the initial growth period, the nutrient solution was removed, and 1 × 10^8^ CFU/mL of the prepared bacterial suspension was added to each jar as the inoculum. Three biological replicates (seedlings) were used per treatment group. At 1 dpi, 5 dpi and 10 dpi, seedlings were removed and rinsed with sterile water. Young root segments, particularly from the root hair zone, were excised and examined microscopically. Colonisation was visualised using green fluorescent protein (GFP)‐labelled strains under a fluorescence microscope, and qualitative differences in fluorescence intensity were used to infer relative colonisation efficiency among the strains.

To quantify the colonisation of *B. subtilis* R31 surfactin hyperproduction strains in banana roots, plants were first rinsed with sterile water. Approximately 2‐cm segments of the root hair zone were excised using sterile scissors. From each plant, 1 g of root material was randomly collected and pooled. The root samples were transferred into a sterile mortar containing 1 mL of phosphate‐buffered saline (PBS), ground gently and the homogenate was transferred into a centrifuge tube containing 8 mL of sterile water. This suspension was allowed to stand for 15 min to facilitate bacterial release and was designated as the ‘mother liquor’. Serial dilutions of the mother liquor were performed using sterile water, and 100 μL of each dilution was spread onto NA plates supplemented with 50 μg/mL erythromycin to select for GFP‐labelled bacterial strains. Three dilution gradients were selected for plating, with each dilution plated in triplicate. Plates were incubated at 37°C for approximately 24 h. CFUs exhibiting green fluorescence under UV light were counted, and the average bacterial population was calculated and expressed as CFU per gram of root tissue.

### 2.9 Transcriptome Analysis of *B. subtilis* R31 Wild Type Versus Surfactin Hyperproduction Strains

To investigate the transcriptomic profiles of *B. subtilis* R31 and its surfactin hyperproduction strains, cultures of WT R31, R31 (*comK* overexpression), R31 (*phrC* overexpression) and R31 (*comK*/*phrC* co‐overexpression) were grown in MSgg liquid medium within 24‐well plates. After 24 h of incubation at 37°C, bacterial cultures were harvested from each condition. Cells were collected by centrifugation at 4°C for 10 min at high speed using 50 mL centrifuge tubes, with three biological replicates per strain. The cell pellets were snap‐frozen in liquid nitrogen and labelled as R31‐24 h, *comK*‐24 h, *phrC*‐24 h and *comKphrC*‐24 h. Samples were stored at −80°C and submitted to Guangzhou Kidio Biotechnology Co. Ltd. for transcriptome sequencing and analysis. Raw sequencing reads were filtered to remove adapter sequences, reads containing more than 10% unknown bases (N), and reads with over 50% of bases having low‐quality scores (Q < 20) (Figure S5, Table S1). High‐quality clean reads were aligned to the reference genome using standard alignment tools, and transcripts were reconstructed using StringTie (Figure S6, Figure S7 and Table S2). The reconstructed transcripts were compared to annotated genomic features using GffCompare. Functional annotation of target genes was conducted using BLAST searches against several databases, including National Center for Biotechnology Information (NCBI: https://www.ncbi.nlm.nih.gov/), KEGG (https://www.kegg.jp/), Gene Ontology (GO: http://www.geneontology.org/) and Eukaryotic Orthologous Groups (KOG). Gene expression levels were quantified as fragments per kilobase of transcript per million mapped reads (FPKM) (Figure S6). Differential gene expression analysis was conducted using DESeq2 (Figure S7), with the Pearson correlation coefficient (*R* > 0.8) used to assess consistency among biological replicates. Log‐transformed normalised read counts were subjected to PCA to visualise sample variance. DEGs were identified using the thresholds log_2_FoldChange ≥ 1 and FDR < 0.05, with FDR values corrected through the Benjamini–Hochberg procedure.

GO and KEGG enrichment analyses of the identified differentially expressed genes were conducted to assess transcriptomic changes between the WT R31 and surfactin‐overexpressing strains. The raw data generated in this study have been deposited in the National Gene Bank Life Big Data Platform (CNGBdb), in the National Gene Bank Sequence Archiving System (CNSA), with the accession number CNP0007061, and the website is https://db.cngb.org/cnsa/.

### 2.10 Efficacy of *B. subtilis* R31 Against *Fusarium* Wilt of Banana

To evaluate the antifungal activity of *B. subtilis* R31 and its surfactin hyperproduction strains, fermentation broth and a surfactin standard solution were applied onto sterile filter paper discs, with four replicates per treatment placed on each plate. Plates were first refrigerated at 4°C to facilitate even diffusion of the compounds, followed by incubation at 30°C for 7 days. Inhibition zones were measured using a vernier calliper to determine antagonistic efficacy against the fungal pathogen. The fermentation broth used for antagonism assays contained approximately 1 × 10^9^ CFU/mL of live bacteria and was diluted to 1 × 10^8^ CFU/mL for downstream applications.

The fungal pathogen strain XJZ2 was retrieved from cold storage (4°C) and cultured on potato dextrose agar (PDA) for 1 week at 30°C. Mycelial plugs were transferred into potato dextrose broth (PDB) and incubated under shaking conditions (30°C, 180 rpm) for another week. Spores were harvested by filtration through sterile gauze, and the filtrate was diluted with sterile water to adjust the spore concentration to 1.0 × 10^6^ CFU/mL.

A pot‐based inoculation model was used to assess the biocontrol potential of *B. subtilis* R31 strains against *Fusarium* wilt in Brazilian banana ( *Musa acuminata* AAA group, Cavendish cultivar). For treatment setup, 50 mL of either WT or sodium alginate‐encapsulated R31 fermentation broth was applied directly around the root zone of banana seedlings, followed by irrigation with sterile tap water. At 5 dpi, the seedlings were subjected to wound inoculation with XJZ2 spores. Lateral roots were gently incised with a sterile scalpel, and the prepared spore suspension was applied at the wound site. Each treatment group consisted of 30 banana seedlings. Disease symptoms were monitored, and assessments were conducted at 50 dpi. Disease incidence and severity were scored based on shoot leaf wilting and browning of the bulb and pseudostem base, according to the following classification system.

Grade 0: Healthy, no browning or tissue damage.

Grade 1: Up to 25% of leaves yellowing or browning of less than 25% of stem tissue.

Grade 3: 25%–50% of leaves yellowing or 25%–50% stem browning.

Grade 5: 50%–90% leaf yellowing or browning in > 50% of stem, with mild wilting.

Grade 7: Full yellowing, plant death, or severe tissue decay.

$$\begin{array}{c} \text{Morbidity rate}\;\left(\mathrm{DI}\right)=(\text{Number of diseased plants}/\text{plant}\;\\ \text{population})\times 100\% \end{array}$$

$$\begin{array}{c} \text{Disease index}\;\left(\mathrm{DS}\right)=(\text{representative value of}\;\mathrm{all}\;\text{levels of}\;\\ \text{strains})/\text{highest representative value of total number of}\;\\ \text{strains}\;\text{investigated}\times 100\% \end{array}$$

$$\begin{array}{c} \text{Prevention effect}\;\left(\mathrm{BE}\right)=\left(\text{pathogen control index}\right)/\text{pathogen}\;\\ \text{control index}\times 100\% \end{array}$$

All data were statistically analysed using one‐way ANOVA followed by Duncan's multiple comparison test, with significance defined at *p* < 0.05. Statistical graphs were generated using GraphPad Prism version 9.0.2.

### 2.11 Effect of R31 With Hyperproduction Strains Regulating Banana Rhizosphere Microbiota

To evaluate the microbial communities associated with *M. acuminata* , both rhizosphere soil and root samples were collected from potted banana seedlings using a standardised inter‐root sampling method. The bulk soil around the banana root system was initially removed with a sterile shovel. The root hair zone, along with tightly adhering soil (approximately 1 mm thick), was excised using sterilised scissors. The excised root–soil composite was transferred into 50‐mL sterile centrifuge tubes containing 20 mL of sterile 0.1 M PBS. Samples were gently shaken on an orbital shaker at 120 rpm at room temperature for 20 min. This process allowed for detachment and collection of rhizosphere soil (approximately 2 g per sample). Roots were then removed using sterile forceps, and the soil‐containing suspension was centrifuged at 6000 × g for 20 min at 4°C. The resulting rhizosphere soil pellet was collected for downstream analyses. For each treatment, six biological replicates were obtained. Three replicates were immediately snap‐frozen in liquid nitrogen and sent for microbial community sequencing, while the remaining three were stored at −80°C as backups. Root samples were similarly processed: approximately 0.2 g of fine roots per replicate was clipped using sterile scissors, transferred into 15 mL centrifuge tubes and snap‐frozen in liquid nitrogen. Three root replicates were sent for sequencing, and three were retained at −80°C for future analysis. Total genomic DNA was extracted from both root and rhizosphere soil samples. The V3–V4 hypervariable regions of the 16SrRNA gene were amplified using the primers 341F (5′‐CCTACGGGNGGCWGCAG‐3′) and 806R (5′‐GGACTACHVGGGTATCTAAT‐3′). PCR products were gel‐purified, quantified with a fluorescence‐based Qubit fluorometer, pooled in equimolar concentrations and ligated to adapters for sequencing library construction. Amplicon libraries were sequenced on the Illumina MiSeq PE250 platform by Guangzhou Quiio Biotechnology Co. Ltd. Bioinformatics analysis of microbial diversity and composition was performed using the Omicsmart analysis platform (Guangzhou Quiio Biotechnology). Alpha and beta diversity metrics were calculated, and taxonomic profiles were generated. Statistical comparisons among treatment groups were conducted using Tukey's honest significant difference (HSD) test, with a significance threshold of *p* < 0.05.

All the microbiome sequencing data have been deposited in NCBI, with the BioProject accession number PRJNA126745, and the website is https://www.ncbi.nlm.nih.gov/bioproject/1062623.

## 3 Results and Analysis

### 3.1 Surfactin Hyperproduction Strain Construction and Validation

This study developed hyperproduction strains of *B. subtilis* R31 by incorporating the P43 promoter, target genes (*comK* and *phrC*), a 6 × His tag and a T7 terminator into the pHT315 vector using Gibson assembly. Three recombinant plasmids (e.g., pHT315‐*comK*, pHT315‐*phrC* and pHT315‐*comKphrC*) were validated through colony PCR using pHT315‐F/R primers, which yielded products of 1349 bp, 893 bp and 1512 bp respectively (Figure S2A–B). Subsequent sequencing confirmed a 100% identity with the reference sequences available in the NCBI database (Table S3).

Western blot analysis demonstrated robust expression of 6 × His‐tagged ComK (28 kDa) and PhrC (15 kDa) in recombinant strains, which was not detectable in wild‐type (WT) controls (Figure S2C). While the intensity of reference protein bands remained consistent across samples, the signal intensity of ComK exhibited variability among the different strains. Quantitative reverse transcription polymerase chain reaction (RT‐PCR) revealed a significant upregulation of both genes in recombinant strains compared to WT controls (log_2_ [relative expression] > 4.5, *p* < 0.01), with PhrC expression surpassing ComK by a factor of 1.8 (Figure S2D). This disparity in expression levels likely accounts for the diminished ComK immunoblot signals, despite equivalent protein loading across samples.

### 3.2 HPLC of R31 Hyperproduction Strain Surfactin, and Quantitative Results

**FIGURE 1:**
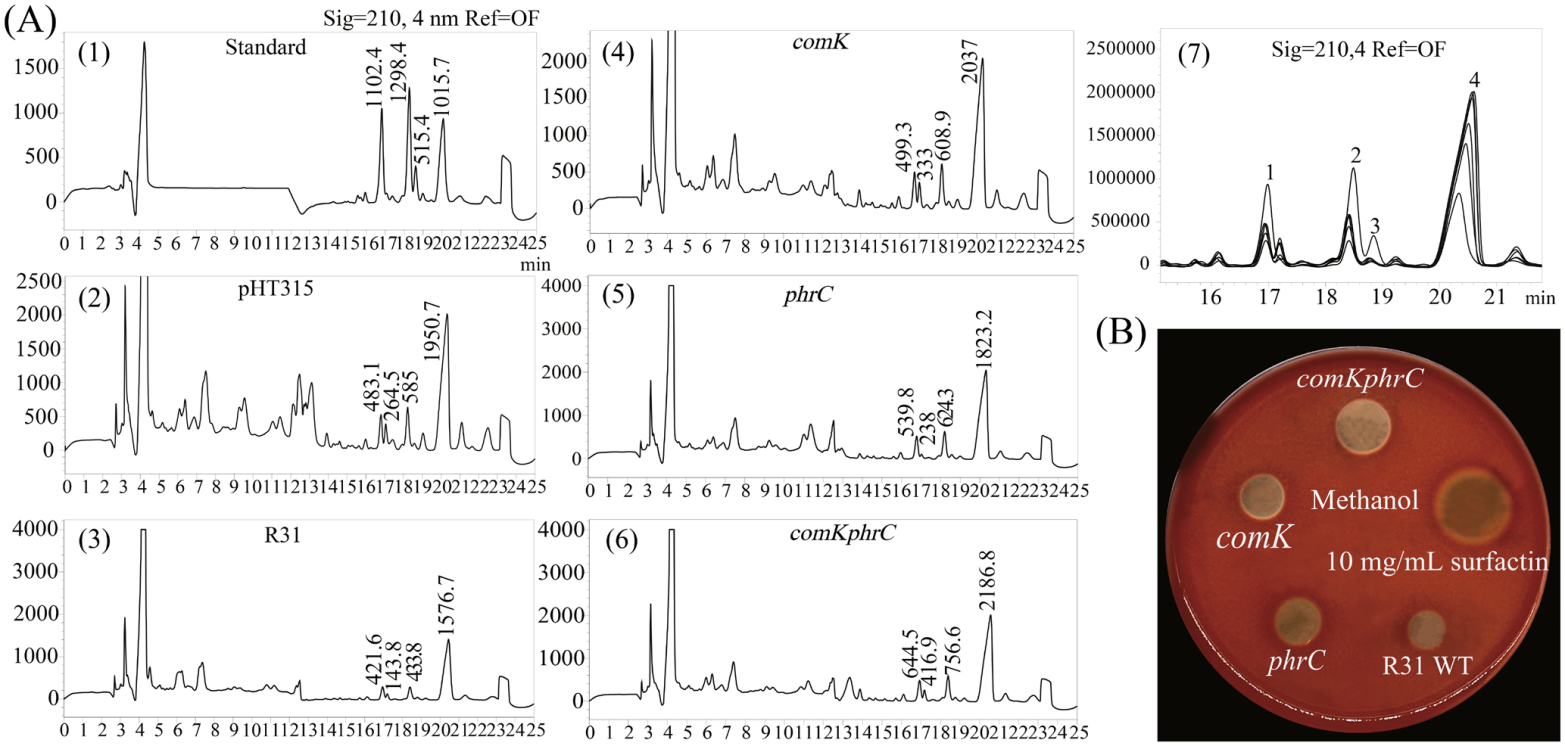
Analysis of surfactin production variability in overexpressing strains; (A): Comparative analysis of surfactin content of R31 and overexpressed strains; (1–6): Surfactant content of each strain of R31 detected by HPLC, *n* = 5; (7): R31 strain and standard samples; (B): Comparison of haemolytic activity of crude R31 lipopeptide extract, and comparison of the differences in surfactin content of each strain, *n* = 3.

**FIGURE 2:**
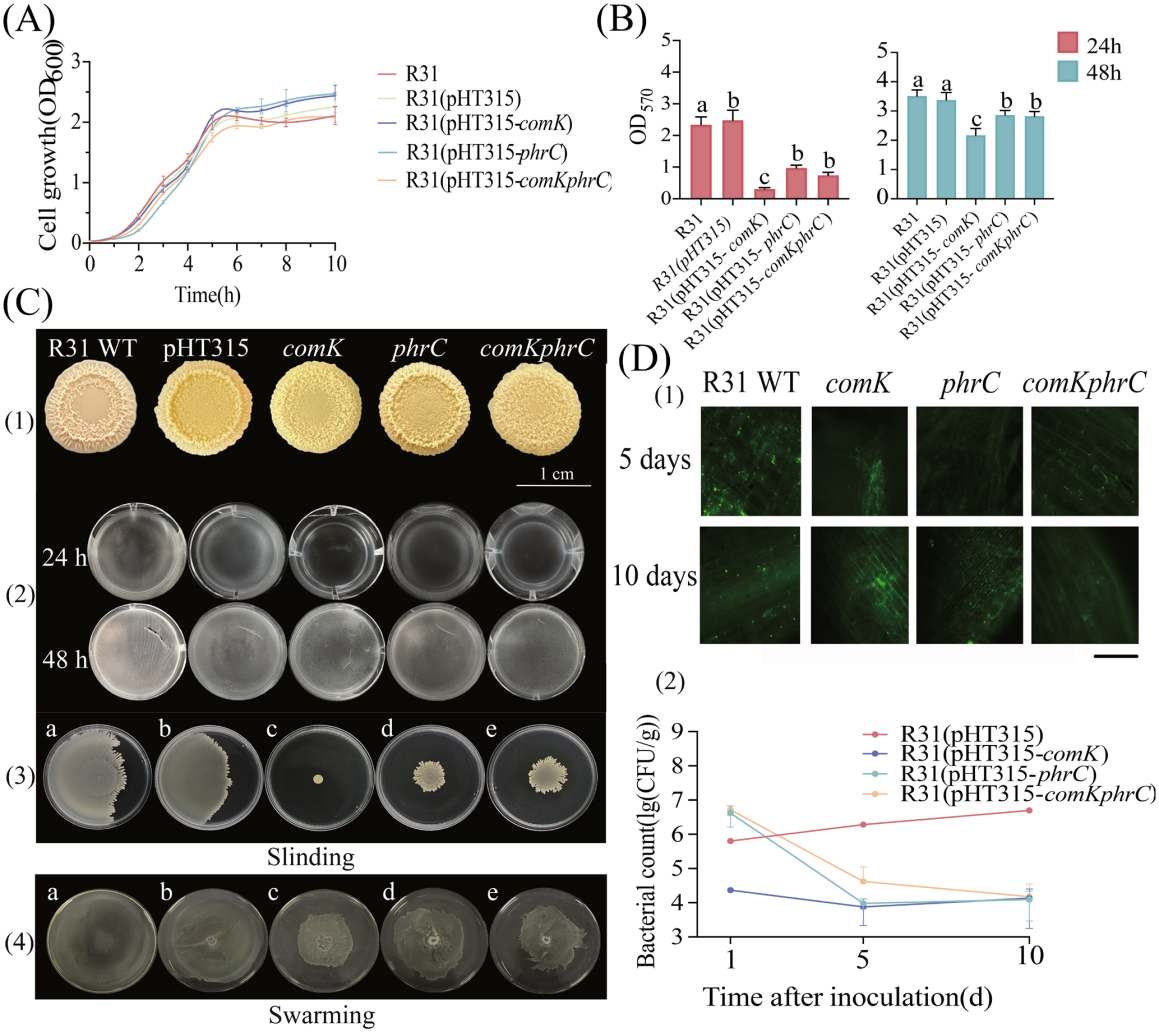
Biological function analysis of R31 surfactin hyperproduction strains; growth of R31 hyperproduction strains contrasted with biofilm formation; (A): Growth curves of R31 wild‐type and surfactin hyperproduction strains, error line is mean ± standard deviation, *n* = 3; (B): Biofilm quantification of R31 wild‐type and surfactin hyperproduction strains, as detected by OD_570_ crystal violet staining in 12‐well plates for biofilm amount. The readings are the results of biofilm quantification. Error lines in the graphs are mean ± standard deviation, *n* = 4; (C): Colony morphology biofilm of R31 wild‐type and surfactin hyperproduction strains cultured on MSgg solid and liquid media for 48 h, *n* = 5; (D): (1) The colonisation ability of R31 wild‐type strain and surfactin‐hyperproduction strain on Musa plantain was observed by fluorescence electron microscopy. Green fluorescent protein (GFP) was transferred to the fluorescence of R31 wild‐type strain and surfactin‐producing strain in the surface cells of banana roots. The magnification was 200×. (2) The colonisation and elimination dynamics of R31 wild type and surfactin‐producing strain in banana roots were measured. The R31 inoculum concentration of each strain was 1 × 10^8^ CFU/mL, the error line in the figure is the mean ± standard deviation, *n* = 3; Observation of motility of R31 wild‐type and surfactin‐producing strains.

**FIGURE 3:**
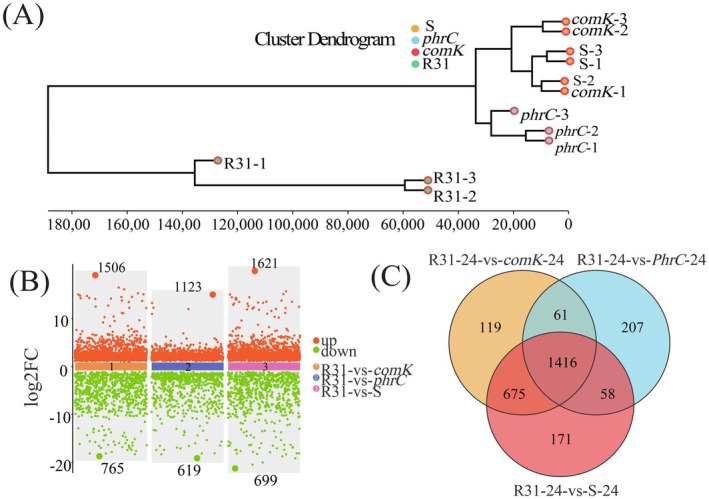
Transcriptome analysis of R31 hyperproduction strains; (A): Using the expression of all genes or the target gene set, hierarchical clustering was carried out for the relationships of all samples, The ordinate represents the Euclidean distance between samples, and each smallest branch of the tree represents a sample. The further down the tree, the closer the sample distance, *n* = 3. (B): Statistics of differential number of genes between R31 wild‐type and overexpressed strains, Orange (up regulated) and green (down regulated) dots indicate differences in gene expression, the judgement criteria were an FDR < 0.05, and the difference is more than two times the multiple, the middle colour block represents no differential expression, *n* = 3; (C): Utilise Venn diagrams to display the number of common target genes among different comparison groups., The screening conditions for significant differential genes are log2FC > 1 and FDR < 0.05.

**FIGURE 4:**
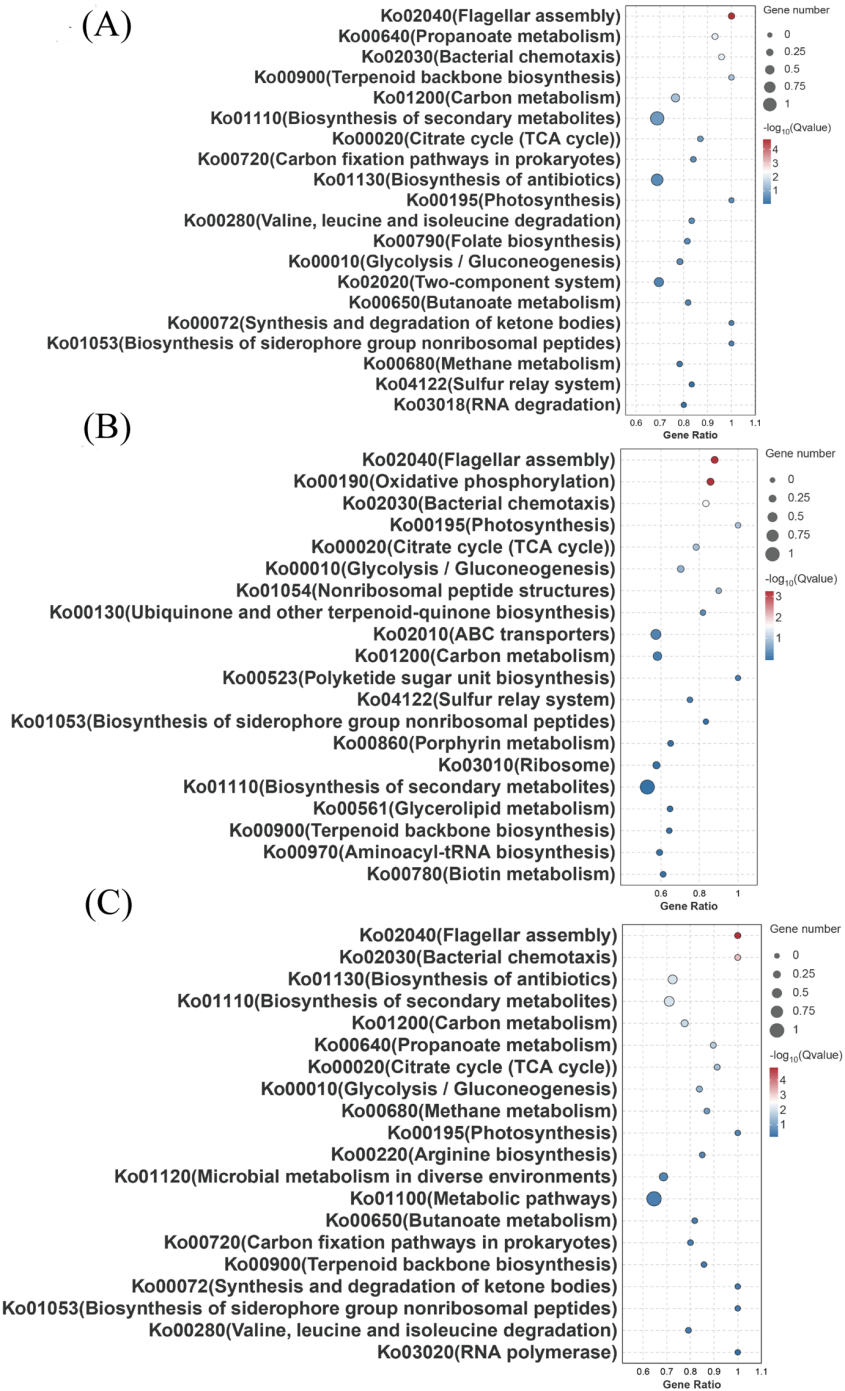
Enrichment analysis of differentially expressed gene pathways in each group; (A, B and C): Bubble plot of KEGG enrichment analysis of differential genes between R31 wild‐type and overexpressed strains RichFactor refers to the ratio of the number of transcripts located in the pathway entry to the total number of transcripts located in the pathway entry among all transcripts in the differentially expressed transcripts, the greater the RichFactor, the greater the RichFactor. Indicates a higher degree of enrichment. q values are *p* values adjusted for multiple hypothesis testing and range from 0 to 1. The closer to zero, the more significant the enrichment. The *q* values of the top 20 paths were sorted from small to large, and *n* = 3 was plotted. Next to it is the coexpression network analysis of each component to observe the effect of R31 and overexpression strain on the expression of regulatory pathways during transcription, cro ≥ 0.5, *p* ≤ 0.05, *n* = 3 for all data analysis.

**FIGURE 5:**
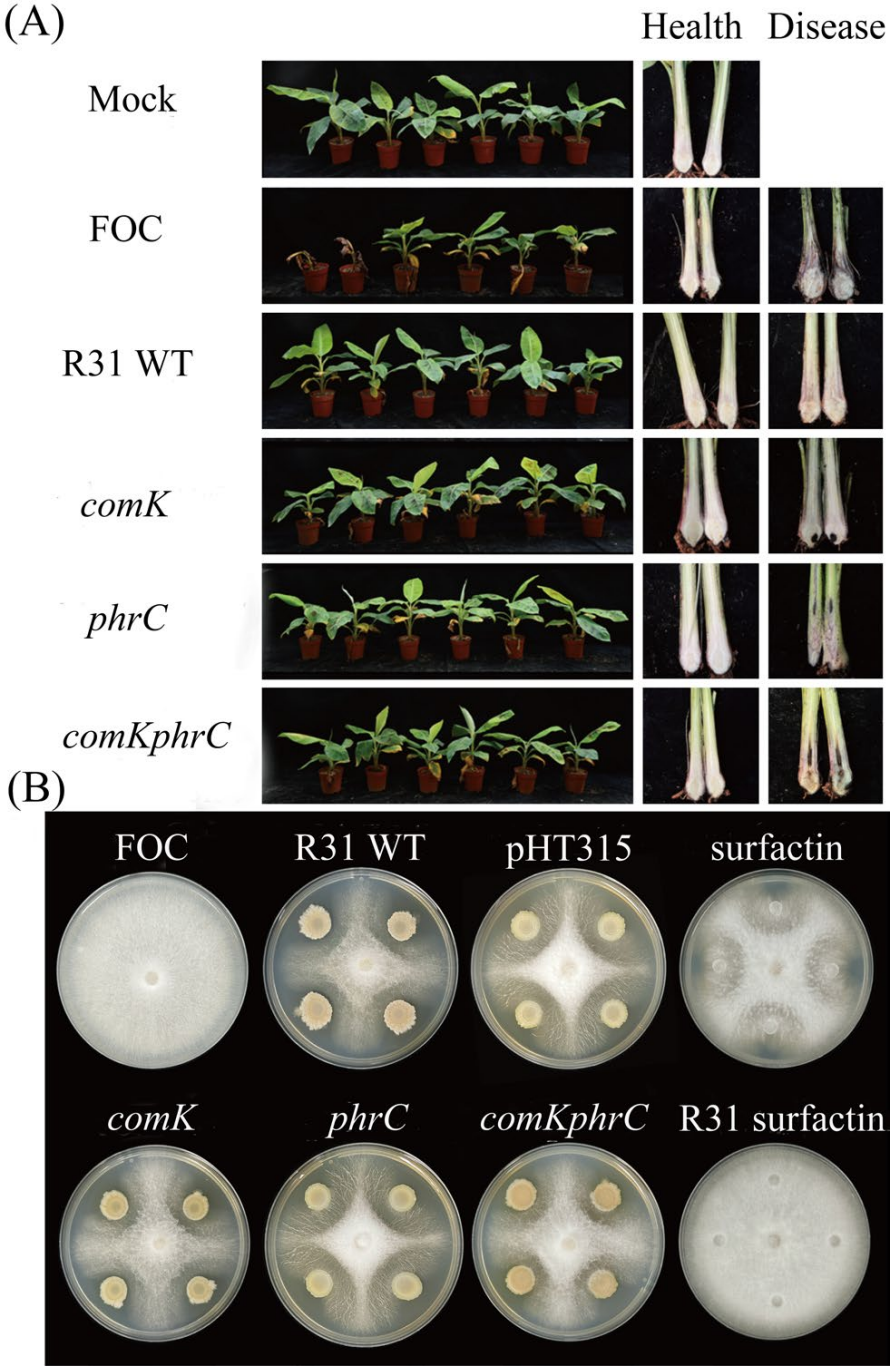
Inhibition of *B. subtilis* R31 wild‐type and surfactin overexpressed strains; (A): R31 wild‐type and surfactin hyperproduction strains against banana blight (50 days) Mock for clear water control, CK for *F. oxysporum* XJZ2 control, *n* = 30. (B): Inhibitory effects of *B. subtilis* R31 wild‐type and surfactin overexpressing strains against *F. oxysporum* XJZ2 (Observation results 7 days after inoculation, CK is *F. oxysporum* XJZ2), *n* = 3.

**FIGURE 6:**
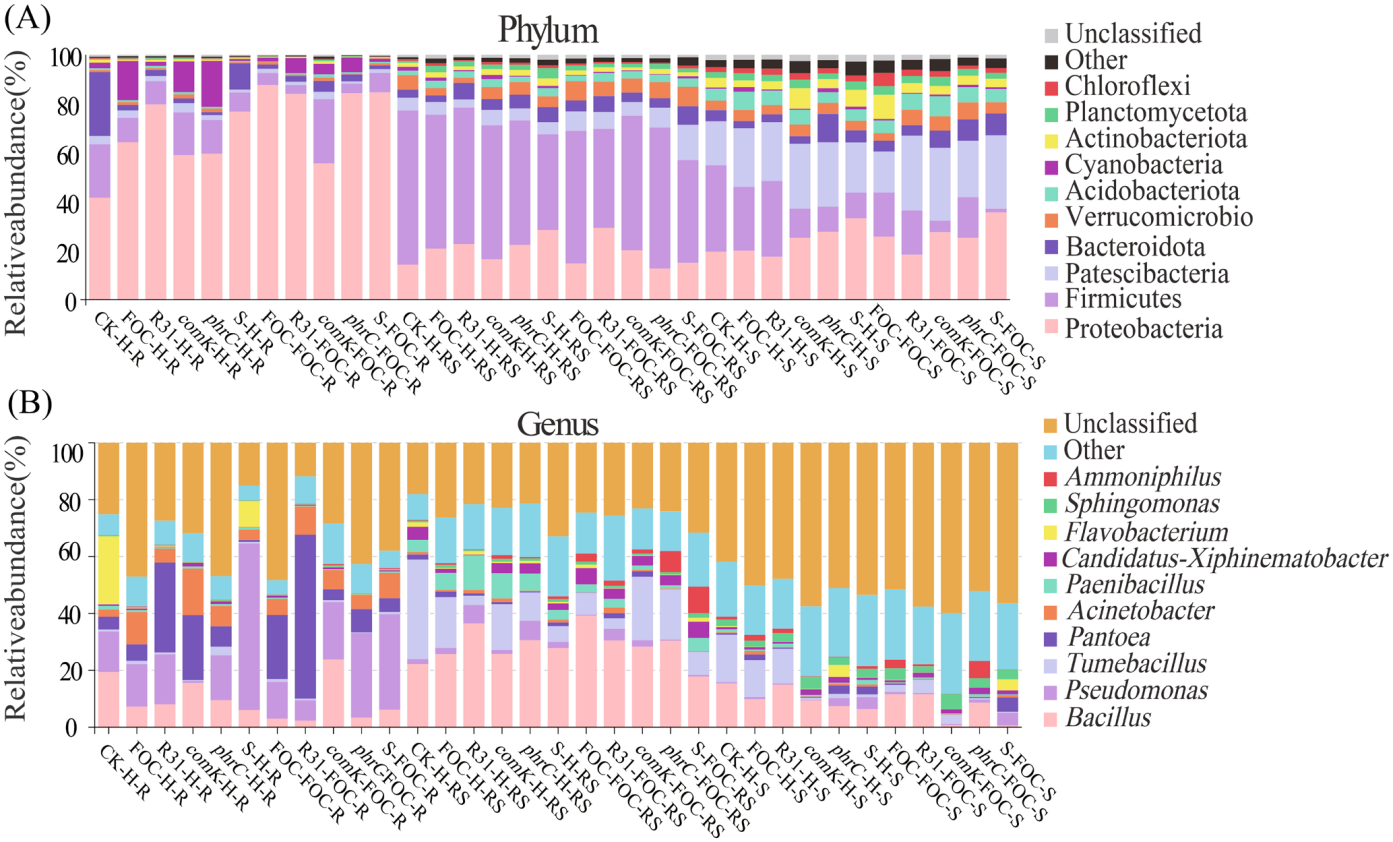
Microbiome analysis of banana plants; the microbial community relationships in each treatment group were compared, and the distribution of microbial flora at the genus level was analysed through stacked plots. By default, the top 10 abundant species at each taxonomic level would be displayed; other known species would be merged into the ‘Other’ category, and unknown species would be labelled as ‘Unclassified’. (1): Phylum classification, (2): Genus classification.

**FIGURE 7:**
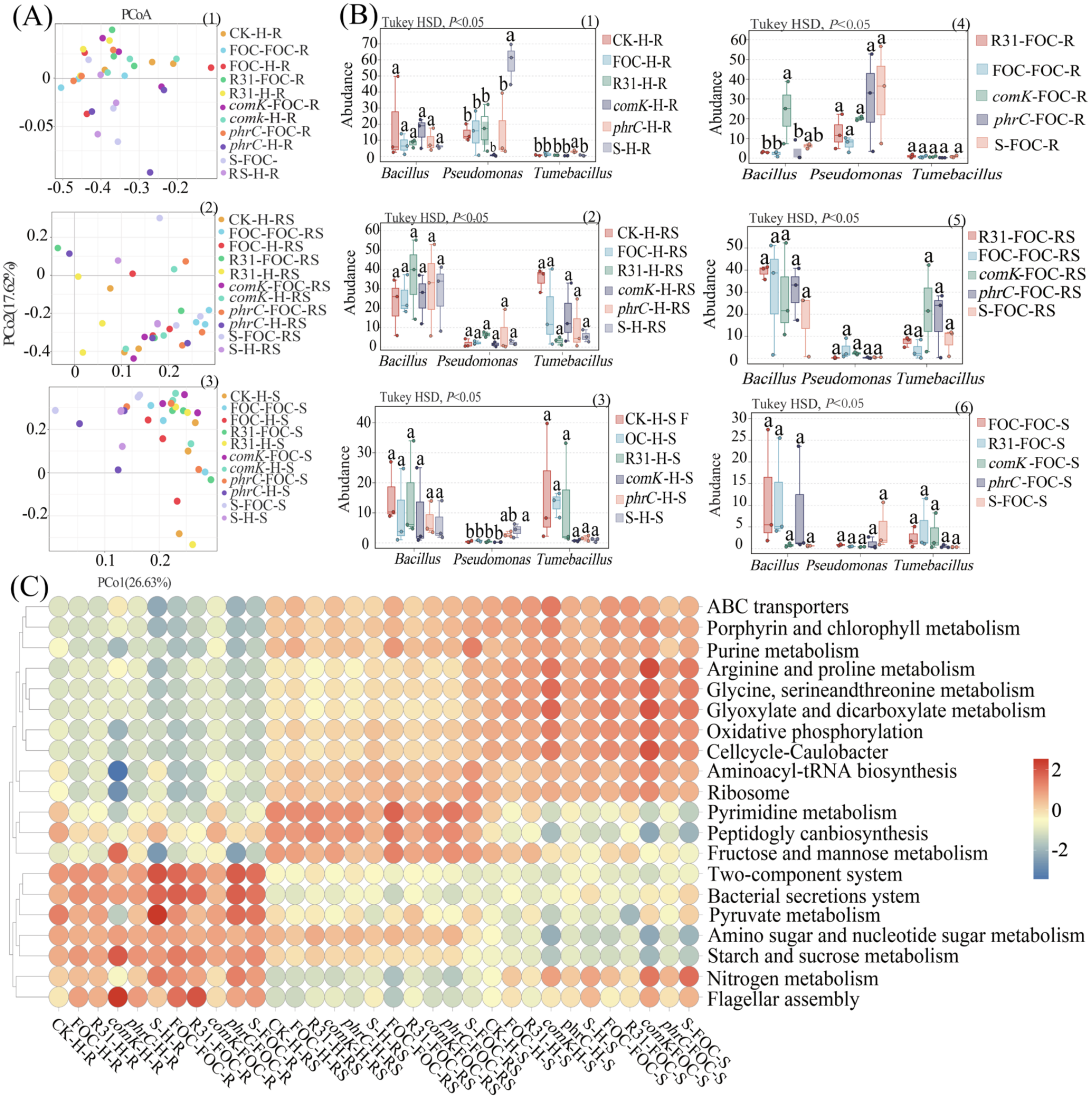
Analysis of the key microbial community structure of banana plants; **(A)** The PCoA analysis results were used to visualise the relationships between root systems, rhizosphere soil and soil, and to evaluate the sample grouping and within‐group repeatability. The more similar the samples are, the closer the distance reflected in the PCoA plot. Additionally, samples from different environments often exhibit their own aggregated distribution characteristics. In this figure, (1) is the community distribution of root system samples in each group, (2) is the community distribution of rhizosphere soil samples and (3) is the community distribution of soil samples. **(B)** After multiple comparisons of differences between multiple groups were conducted using the Turkey HSD method, the top three abundant genera were selected for multiple comparisons. (1–3) are diseased roots, root system soil and soil samples, (4–6) are healthy roots, rhizosphere soil and soil samples. Each sample was repeated three times. **(C)** Functional abundance heatmaps were used to conduct functional analysis of healthy and diseased samples. This is a graphical display that represents functional relative abundance through colour gradients and clusters based on functional abundance similarity, while incorporating grouping information of sample treatments or sampling environments. It can visually display the functional clustering of samples with the same treatment or similar environments under KEGG Level 3, and reflect the similarities and differences in KEGG functions of each sample or group. The Tax4Fun functional abundance heatmaps default to displaying the top 20 functions. In all charts, the sample repetition times are *n* = 3, *p* < 0.05.

Surfactin consists of C13–C15 fatty acid homologues, which were separated using high‐performance liquid chromatography (HPLC) and identified as four peaks with retention times between 16 min and 21 min in extracts from *B. subtilis* R31 (Figures 1A–1, Figures 1, 2, Figures 1, 2, 3, Figures 1, 2, 3, 4, Figures 1, 2, 3, 4, 5, Figures 1, 2, 3, 4, 5, 6). A comparative analysis with standard samples confirmed the predominance of C13–C15 homologues. Notably, hyperproducing strains demonstrated an increase in surfactin‐4 levels and a decrease in surfactin‐3 levels compared to the WT, while the levels of surfactin‐2 remained unchanged (Figures 1, 2, 3, 4, 5, 6, 7).

Lipopeptides produced by *Bacillus* (surfactin, iturin, fengycin) show significant haemolytic activity. Haemolysis test is often used to characterise biosurfactant production (Ambrin et al. 2018; Mnif et al. 2016). Studies show surfactin's haemolytic activity is directly related to its biosurfactant production capacity, and can be an indicator for lipopeptide production like surfactin. *β*‐haemolytic assays demonstrated that surfactin induces erythrocyte lysis, with hyperproducing strains exhibiting lytic zones that were 1.8 times larger than those of the WT (Figure 1B), which is consistent with the quantification results obtained from HPLC. Control experiments using the empty pHT315 vector indicated minimal differences in production levels.

The hyperproduction of dual *comK/phrC* resulted in a maximised yield of surfactin, demonstrating a 45% increase compared to the WT. However, the concentrations obtained, despite this enhancement, were still insufficient to improve biocontrol efficacy (Table 1). Next, we need to verify what effects the overexpression of surfactin has on the physiological morphology of the strain.

**TABLE 1:** Calculation of surfactin yield.

Strain name	Surfactin yield (mg/L)
R31 WT	3.36 ± 0.49c
R31 (pHT315)	3.79 ± 0.15bc
R31 (pHT 315‐*comK*)	4.13 ± 0.19b
R31 (pHT315‐*phrC*)	4.84 ± 0.11a
R31 (pHT315‐*comKphrC*)	4.86 ± 0.53a

*Note:* The surfactin production in the five treatment groups (including WT) was all normally distributed. Through Duncan's multiple range test, different lowercase letters within the same column indicated statistically significant differences, with *p* < 0.05. Each sample was subjected to three biological replicates. The statistical results showed that overexpression of *phrC* and *comK* could both increase surfactin production, and the yield of double overexpression of *comK/phrC* could reach 45%.

### 3.3 Biological Morphology and Behavioural Effects of Overexpressed Strain R31

By obtaining the WT R31 strain and the R31 strain with high surfactant production ability, and verifying the differences in surfactant content, it was found that the R31 strain with dual overexpression of *comK/phrC* was a high‐producing strain of surfactin. To further verify the impact of surfactin on the function of the strain, we conducted biological phenotypic tests to compare the differences of the strains. The results showed a similar growth pattern, characterised by logarithmic growth from 2 h to 5 h, followed by stabilisation. However, the WT R31 had a slightly faster growth rate during the logarithmic growth phase, but the strain with excessive surfactin production eventually reached a higher cell density (Figure 2A).

Biofilm formation was observed in *B. subtilis* R31 on MSgg solid plates after 72 h (Figures 1C–1). The R31 strains carrying the plasmids pHT315‐*comK* and pHT315‐*comKphrC* exhibited flatter capsules with less pronounced bulges compared to the WT R31 and the R31 strain with the plasmid pHT315‐*phrC*. This difference is likely attributable to the hyperproduction of *comK*, which appears to affect the structural integrity of the biofilm. In liquid MSgg medium, all R31 strains developed a thin pellicle after 24 h, with the WT R31 forming the most substantial biofilm. Biofilm formation was most pronounced in the WT R31 at 48 h. However, it did not develop a solid three‐dimensional structure as observed on solid surfaces (Figure 2C‐2). Quantitative analysis using crystal violet staining indicated that the WT R31 formed biofilms at a faster rate and exhibited the thickest biofilm coating. In contrast, the biofilm formation capabilities of the hyperproduction strains were weaker, with the most significant disparity noted at 24 h. By 48 h, the difference in thickness diminished, with the R31 (pHT315‐*phrC*) strain showing the thickest biofilm among the hyperproduction strains, although it remained less thick than the WT. Notably, surfactin hyperproduction was found to delay biofilm formation on liquid surfaces and resulted in a reduction in pellicle thickness ranging from 18% to 39%. The R31 (pHT315‐*comK*) strain exhibited the most significant inhibition of biofilm formation, likely due to the effects of *comK* hyperproduction on biofilm development (Figure 2B).

The observation of bacterial diffusion on agar plates demonstrated that the WT *B. subtilis* R31 exhibits robust motility, successfully covering the entire surface of 0.5% nutrient agar (NA) semisolid plates within 8 h, and approximately 80% of the surface of 0.7% Luria–Bertani (LB) semisolid plates within 24 h. The swimming and sliding capabilities of both the WT R31 and the R31 (pHT315) strains were found to be comparable, suggesting that the introduction of the pHT315 empty vector did not significantly impact motility (Figures 2, 3). In contrast, the three strains exhibiting hyperproduction of surfactin demonstrated a marked reduction in swimming ability and a substantial decrease in sliding ability. Specifically, the strains R31 (pHT315‐*phrC*) and R31 (pHT315‐*comKphrC*) showed equivalent motility, while R31 (pHT315‐*comK*) exhibited slightly diminished motility compared to the former two and showed no sliding ability. These results indicate that the hyperproduction of surfactin primarily impairs the motility of *B. subtilis* R31, likely through the upregulation of the *comK* gene, which promotes surfactin synthesis (Figures 2, 3, 4).

In root colonisation assays, the WT strain R31 predominantly attached to root hairs on Day 1, with no green fluorescence detected on the surface cells of the roots. By Day 5, WT R31 had established robust biofilms, exhibiting bright green fluorescence on the root surface cells. In contrast, the hyperproduction strains demonstrated weak and sporadic fluorescence, accompanied by diminished colonisation. All hyperproduction strains showed similar colonisation patterns, consistently yielding lower levels of colonisation compared to the WT. Dilution plating analyses indicated that the colonisation of WT R31 increased over time, whereas the hyperproduction strains exhibited a decline, with WT R31 demonstrating significantly stronger colonisation overall. Following inoculation at a concentration of 1 × 10^8^ CFU/mL, all strains were detected within the roots. However, R31 had not yet reached the banana root epidermal cells. The R31 (pHT315‐*comK*) strain exhibited the least colonisation, likely attributable to its impaired swimming ability. The disparity in colonisation success between the WT and the overexpressed R31 strains expanded by Day 5. The success of R31 colonisation was positively correlated with its biofilm formation capability, which was notably weaker in the hyperproduction strains (Figure 2D‐1). Consequently, despite the presence of R31 bacteria in the banana roots, only a limited number successfully established colonisation. WT R31 colonisation peaked and stabilised after 10 days, while the colonisation levels of the hyperproduction strains either slightly increased or decreased, ultimately stabilising at 1.3 × 10^4^ CFU/g (Figure 2D‐2).

### 3.4 Transcriptomic Analysis of Surfactin‐Hyperproduction *B. subtilis* R31 Strains

WT and surfactin‐hyperproducing strains of *B. subtilis* R31 initiated population swimming and biofilm formation within 24 h, with surfactin synthesis commencing during the logarithmic growth phase. Therefore, transcripts collected at 24 h were selected for differential gene expression analysis to identify genes exhibiting a false discovery rate (FDR) of less than 0.05 and an absolute log_2_ fold change (|log_2_FC|) greater than 1. The expression levels of all known genes in each sample were analysed using principal component analysis (PCA) and correlation heatmap analysis, using R language packages (Figure S3). Hierarchical clustering of the relationships among all samples, based on the expression levels of all genes or specific target gene sets, provides insights into the relationships between samples. This approach aids in assessing the repeatability of the samples and identifying potential outliers (Figure 3A).

PCA revealed that the first two principal components (PC1 and PC2) accounted for 88% of the total variance, suggesting a high degree of reproducibility among the samples, with no outliers and significant differences observed between the groups. Correlation analysis of gene abundance further confirmed the repeatability of the samples and indicated a high level of transcriptional consistency. Incubation in MSgg medium for 24 h resulted in the identification of 2271 differentially expressed genes (DEGs) in the *comK* strain (1506 upregulated and 765 downregulated), 1742 in the *phrC* strain (1123 upregulated and 619 downregulated) and 2320 in the *comKphrC* strain (1621 upregulated and 699 downregulated) (Figure 3B), A total of 1416 differentially expressed genes were screened by Venn diagram (Figure 3C).

The annotation and classification of genes within the Kyoto Encyclopedia of Genes and Genomes (KEGG) database revealed their distribution across various metabolic and signalling pathways. Following the application of multiple testing correction with a Q‐value threshold of 0.05, a total of 23 significantly enriched pathways were identified, which were categorised into five classes. The pathways exhibiting the highest levels of enrichment included global/overview maps, encompassing 469 genes, and carbohydrate metabolism, which involved 156 genes. The KEGG enrichment analysis provided insights into the differences observed in the hyperproduction of *comK/phrC*, surfactin synthesis, biofilm formation and population swimming (Figure S4).

DEGs were significantly enriched in pathways related to flagellar assembly, bacterial chemotaxis, carbon metabolism, the tricarboxylic acid (TCA) cycle, glycolysis and secondary metabolite synthesis (*p* < 0.05). The R31 (pHT315‐*phrC*) and R31 (pHT315‐*comK*) groups showed enrichment characteristics in the ATP‐binding cassette (ABC) transporter pathway (Ko02010) and secondary metabolite synthesis (Ko01110) (Figure 4A,B), while the R31 (pHT315‐*comKphrC*) group showed enrichment characteristics in the metabolic pathway (Ko01110), which was consistent with the highest yield of surfactant produced by this strain among the high‐yield strains. In addition, the amino acid metabolism pathway was significantly enriched, providing necessary precursors for cell growth and surfactant synthesis (Figure 4C).

The research identified key genes in *Bacillus* associated with flagellar assembly and biofilm formation for analysis using Real‐time PCR, subsequently validating the transcriptome data. The results indicated that among the DEGs in the R31 wild type, *comK* hyperproduction strains, *phrC* hyperproduction strains and *comKphrC* hyperproduction strains, the expression of the *tasA* gene was downregulated by factors of 2.67, 0.18 and 2.28 respectively. Similarly, the *epsA* gene exhibited downregulation by factors of 2.59, 0.54 and 1.92. *bslA* hydrophobic proteins are implicated in the three‐dimensional structure and hydrophobicity of biofilms. In the *comK* and *comKphrC* hyperproduction strains, the expression of the *bslA* gene was downregulated by 1.92 and 1.37 times, respectively, whereas in the *phrC* hyperproduction strains, it was upregulated by a factor of 0.72. The downregulation of these genes suggests that biofilm formation is inhibited due to the reduction of extracellular proteins, extracellular polysaccharides and hydrophobic proteins at 24 h. The alignment of the *qPCR* results with the transcriptome differential expression trends further substantiates the reliability of the transcriptome results (Table 2).

**TABLE 2:** DEGs in biofilm metabolic pathways.

Genes	R31 (pHT 315‐*comK*)		R31 (pHT315‐*phrC*)		R31 (pHT315‐*comKphrC*)	
	log2 (FC)	log2 (RT)	log2 (FC)	log2 (RT)	log2 (FC)	log2 (RT)
*tasA*	−2.67	−6.21	−0.18	−2.72	−2.28	−5.15
*tapA*	−0.36	—	−0.80	—	−0.59	—
*epsA*	−2.59	−4.36	−0.54	−0.87	−1.92	−3.09
*bslA*	−1.92	−4.94	0.72	−0.9	−1.37	−5.64
*abrB*	2.21	—	2.19	—	3.01	—
*sinI*	12.17	—	9.95	—	12.6	—
*sinR*	−1.06	—	−1.12	—	−0.90	—
*spo0A*	−1.97	—	−1.35	—	−1.15	—

*Note:* Using the empty vector pHT315 as the reference (log2FC = 0), the table presents the transcriptional changes of eight membrane regulatory genes in three overexpression backgrounds (*comK*, *phrC*, *comKphrC*). FC represents the relative expression fold obtained from qRT‐PCR or RNA‐seq, and |log2FC| ≥ 1 is considered as a significant upregulation or downregulation (*p* < 0.05, after FDR correction). Each test sample was biologically replicated three times, and all data are the average values (*n* = 3).

Based on the results of the transcriptome analysis, we hypothesised that *comK* is the dominant factor regulating the expression of genes related to biofilms. Its overexpression significantly inhibited the synthesis genes of the matrix (*tasA, epsA, bslA*) and activated the inhibitory factor *abrB*; *phrC* had a limited effect on the transcriptional profile and did not show significant synergy with *comK*. This transcriptional pattern explains why surfactin production increased by 45% but did not lead to an increase in biocontrol efficacy—the limited formation of biofilms may have weakened the strain's colonisation and stress resistance capabilities on plant surfaces.

### 3.5 Evaluation of Surfactin's Biocontrol Efficacy Against *F. Oxysporum* XJZ2

Laboratory assays demonstrated that a crude extract of surfactin at a concentration of 10 mg/mL resulted in the thinning of hyphae. However, it did not inhibit the spread of the organism, indicating that a higher concentration of surfactin is necessary for effective inhibition. The analysis of antibacterial rates revealed that the strain R31 (pHT315‐*comK*) exhibited the highest inhibition rate at 51.84%, whereas R31 (pHT315‐*comKphrC*) showed the lowest inhibition rate at 49.45% (Table S4). These results suggest that surfactin alone is not sufficient to effectively inhibit *F. oxysporum* XJZ2 and may instead play a role in the regulation of signalling molecules in the R31 strain (Figure 5B).

Field efficacy assessments conducted using potted plants demonstrated that inoculation with XJZ2 resulted in leaf yellowing and plant mortality approximately 50 dpi. The incidence and severity of the disease were highest in the group treated with *F. oxysporum* XJZ2, exhibiting an incidence rate of 46.67% and a severity rate of 24.76% (Figure 5A). Among the various treatments, R31 (pHT315‐*comK*) proved to be the most effective, with an incidence rate of 6.67%, while R31 (pHT315‐*comKphrC*) exhibited a higher incidence rate of 13.33%. ANOVA analysis indicated no significant differences in biocontrol rates at *p* < 0.05. Notably, the strain with the highest surfactin production exhibited the lowest biocontrol rate, suggesting that surfactin may play a dual regulatory role in the biocontrol efficacy of R31 (Table S5).

### 3.6 Microbial Community Dynamics in Banana Rhizosphere Under *F. Oxysporum* and *B. subtilis* R31 Treatment

After filtering, assembling and clustering the original data, the ‘Effective Tags’ were obtained for the OUT (Operational Taxonomic Units) abundance statistics. On average, each treatment group obtained 103,489 Tags, among which 65,593 (mean) were Tags with species annotations. The Tag sequences were clustered based on similarity and divided into 2310 (mean) different classification sets (Figure S8, Tables S6–S7). Alpha diversity indices analysis revealed different microbial patterns, as presented in Table S4. In healthy banana roots, inoculation with *F. oxysporum* XJZ2 (FOC) significantly reduced phylogenetic diversity (PD) across treatments. The application of the R31 (pHT315‐*comK*) strain resulted in notable declines in both species richness, as indicated by the Chao1 index (385 ± 21 compared to 512 ± 34 in the control, *p* < 0.01), and PD whole tree (28.4 ± 2.1 versus 35.7 ± 3.4, *p* < 0.05), while species evenness remained unaffected (Table S8). Conversely, the application of R31 restored microbial richness in diseased roots, with R31 (pHT315‐*comK*) demonstrating significant increases in both richness (Chao1: +42%) and PD (+38%) when compared to FOC‐infected controls. The alpha diversity indices of rhizosphere and bulk soil microbiomes remained stable across treatments, suggesting that R31‐mediated selection preferentially enriched beneficial taxa while suppressing pathogenic or neutral species without altering overall diversity metrics (Figure S9, Tables S9–S10). Root endosphere analysis demonstrated treatment‐dependent dominance patterns. Control roots were predominantly composed of Flavobacterium (23.93%) while FOC/R31 treatments shifted dominance to *Pantoea* and *Pseudomonas* (Figure 6). Principal Coordinates Analysis (PCoA) utilising unweighted UniFrac distances revealed treatment‐specific clustering patterns (Figure 7A). In healthy plants, R31 (pHT315‐*phrC*) treatments maintained rhizosphere communities that closely resembled controls (Bray–Curtis similarity > 75%), whereas other treatments induced significant divergence. The microbiomes of diseased roots exhibited partial overlap between FOC and R31 treatments, indicating shared features of dysbiosis. The ANOSIM analysis based on the unweighted UniFrac distance matrix results showed that all treatment combinations significantly altered the bacterial community structure (*p* = 0.001). Among them, the inter‐group differences in the healthy rhizosphere soil were the most prominent (*R* = 0.8667), followed by the diseased rhizosphere soil (R = 0.7081), indicating that the rhizosphere microenvironment was the most sensitive to the application of different strains (R31, *comK*, *phrC*, *comKphrC*). It is noteworthy that the R values of the healthy group at the same sampling site were generally higher than those of the diseased group, suggesting that the infection of pathogen FOC may have weakened the significance of the community differences between different treatments (Table 3). Successful colonisation by R31 correlated with an increased abundance of *Bacillus* (15.57%–23.77%), particularly in strains exhibiting hyperproduction of *comK*. Notably, the disease‐suppressive R31 (pHT315‐*comK*) maintained higher levels of *Bacillus* (23.77% compared to 8.42% in FOC) despite the presence of the pathogen. Rhizosphere communities exhibited dominance by *Tumebacillus* (35.01%) in controls, which was replaced by *Bacillus* and *Paenibacillus* under R31 treatments. Disease progression was associated with a decrease in the abundance of the functional consortium (*Bacillus* + *Tumebacillus* + *Paenibacillus*: 61.48% in healthy vs. 38.92% in diseased) (Figure 7B). Tax4Fun analysis revealed compartment‐specific metabolic shifts (Figure 7C). Root endospheres exhibited upregulation of flagellar assembly (ko02040) and nitrogen metabolism (ko00910), particularly in surfactin‐hyperproducing R31 strains. Diseased rhizospheres showed suppressed nitrogen metabolism but elevated peptidoglycan biosynthesis (ko00550). R31 treatments restored metabolic profiles to levels approaching those of the controls across all compartments.

**TABLE 3:** Results of ANOSIM analysis based on unweighted UniFrac distance.

Group	R‐value	*P*
Healthy root group		
CK‐H‐R vs. FOC‐H‐R vs. R31‐H‐R vs. *comK*‐H‐R vs. *phrC*‐H‐R vs. *comKphrC*‐H‐R Healthy rhizosphere soil group	0.8667	0.001
CK‐H‐RS vs. FOC‐H‐RS vs. R31‐H‐RS vs. *comK*‐H‐RS vs. *phrC*‐H‐RS vs. *comKphrC*‐H‐RS Healthy soil group	0.6691	0.001
CK‐H‐S vs. FOC‐H‐S vs. R 31‐H‐S vs. *comK*‐H‐S vs. *phrC*‐H‐S vs. *comKphrC*‐H‐S Pathogenic root group	0.6609	0.001
FOC‐FOC‐R vs. R31‐FOC‐R vs. *comK*‐FOC‐R vs. *phrC*‐FOC‐R vs. *comKphrC*‐FOC‐R Pathogenic rhizosphere soil group	0.7081	0.001
FOC‐FOC‐RS vs. R31‐FOC‐RS vs. *comK*‐FOC‐RS vs. *phrC*‐FOC‐RS vs. *comKphrC*‐FOC‐RS Pathogenic soil group	0.6296	0.001
FOC‐FOC‐S vs. R31‐FOC‐S vs. *comK*‐FOC‐S vs. *phrC*‐FOC‐S vs. *comKphrC*‐FOC‐S	0.6326	0.001

*Note:* The unweighted UniFrac distance matrix was used for the overall community difference test. The ANOSIM was employed to compare the effects of different treatments (CK, FOC, R31, *comK*, *phrC*, *comKphrC*) on the bacterial community of roots (R), rhizosphere soil (RS) and nonrhizosphere soil (S) in both healthy and diseased states. The R values in the table represent the degree of difference between the comparison groups. *R* > 0.75 indicates a significant difference; *R* > 0.5 indicates a moderate difference; and *R* > 0.25 indicates a small difference.

## 4 Discussion

The hyperproduction of quorum sensing‐regulated transcriptional regulators *comK* and sporulation CSF (*phrC*) in *B. subtilis* R31 significantly enhanced surfactin production, with the *comKphrC* dual‐hyperproduction strain achieving a 45% increase in yield compared to the WT strain. Notably, this hyperproduction of surfactin did not compromise vegetative growth. However, it induced pronounced pleiotropic effects on bacterial social behaviours and ecological interactions. While surfactin is traditionally associated with the promotion of flagellar motility through the upregulation of *hag* and *SwrA*(Ghelardi et al. 2012; Stannius et al. 2025), the research results paradoxically indicate that sustained high‐level surfactin synthesis in engineered strains resulted in a reduction of swarming motility by 18%–39% and impaired biofilm formation. This apparent contradiction may stem from energy trade‐offs between surfactin biosynthesis and the maintenance of motility apparatus, in addition to surfactin‐mediated disruption of stable cell aggregation (Lopez et al. 2009; Wu et al. 2025). Transcriptomic analysis revealed a concurrent suppression of extracellular matrix components (e.g., polysaccharides, γ‐PGA and *aprE* protease), which are essential for biofilm maturation. This suggests that surfactin overproduction triggers a metabolic shift that favours dispersal over the development of sessile communities (Arnaouteli et al. 2021; Zhang et al. 2022).

The ecological consequences of surfactin dysregulation were prominently observed during the colonisation of banana roots. The WT strain R31 demonstrated superior persistence in the rhizosphere, achieving a concentration of 1.3 × 10^4^ CFU/g at 10 dpi, in comparison to hyperproducing strains. This result is consistent with previous reports indicating that chemotaxis‐driven motility becomes secondary to collective swarm movement during surface colonisation (Connelly et al. 2004; Zhou et al. 2016). Although surfactin is recognised as a public good that facilitates the exploitation of nonproducers (Kraigher et al. 2022), the microbiome analyses indicated that a moderate elevation of surfactin in R31 (pHT315‐*comK*) enhanced the efficacy of endophytic colonisation through kin‐selective recruitment. Conversely, the excessive production of surfactin by the *comKphrC* strain was associated with a simplification of the rhizosphere community and a reduction in disease suppression. This underscores the existence of optimal surfactin thresholds for microbiome engineering. These results align with emerging paradigms of surfactant‐mediated kin recognition in *Bacillus* (Jautzus et al. 2022; Lyons and Kolter 2017), where the dynamics of spatial competition balance the cooperative benefits against the metabolic costs.

The global metabolic effect of surfactin extends beyond quorum sensing to include the regulation of carbon catabolism. The observed reduction in growth rate of the WT strain R31 during the logarithmic phase suggests an evolved strategy aimed at mitigating the costs associated with surfactin biosynthesis. This observation aligns with the survival dependencies of *B. amyloliquefaciens* on surfactin‐mediated regulation of carbon flux (Su et al. 2024; Xia and Wen 2023). Furthermore, strain‐specific variations in the relationships between biofilm formation and surfactin production (Therien et al. 2020; Vlamakis et al. 2013) underscore the necessity for context‐dependent characterisation of surfactant functions across different *Bacillus* ecotypes (Aloo et al. 2019; Wu et al. 2022).

From an applied perspective, the results elucidate significant trade‐offs in the engineering of biocontrol strains. While the hyperproduction of surfactin enhances direct antifungal activity, excessive yields may adversely impact root colonisation efficiency through: (1) dysregulation of motility and biofilm formation, (2) suppression of protease synthesis (specifically, downregulation of *aprE*) and (3) destabilisation of protective rhizosphere microbiomes. The superior disease suppression observed in moderate surfactin producers likely reflects a balanced activation of both direct antimicrobial effects and indirect resistance mechanisms mediated by microbiomes, particularly involving the synergism between *Sphingomonas–Bacillus* (Wang et al. 2024; Yuan et al. 2015). Future optimisation of strains should prioritise a systems‐level integration of surfactin signalling with the regulation of the extracellular matrix to sustain ecological fitness while maximising metabolite output.

## 5 Conclusion

*B. subtilis* suppresses plant pathogens through surface competitiveness, a strategy involving the secretion of bioactive metabolites that facilitate colony expansion, ecological niche occupation and biofilm formation to enhance environmental resilience. Certain strains employ a ‘kin recognition’ mechanism to recruit cooperative partners in combating phytopathogens, with surfactin serving as a critical signalling molecule. This study demonstrated that moderate levels of surfactin optimally enhanced the soil competitiveness and biocontrol efficacy of *B. subtilis* R31. However, surfactin hyperproduction led to altered cellular behaviours driven by subpopulation differentiation, disrupting key cooperative traits such as biofilm formation and motility. These results raise important questions regarding the mechanisms by which surfactin mediates interstrain cooperation within the rhizosphere, the identity of microbial partners involved and the resulting impact on soil microbial community structure. Further investigation is needed to elucidate the adaptive strategies underpinning surfactin's role in regulating bacterial behaviour and orchestrating complex multicellular interactions in plant‐associated environments.

## Author Contributions

**Hao‐Jun Chen:** investigation, methodology, formal analysis, writing – original draft, conceptualization, validation, data curation, visualization, writing – review and editing, project administration. **Yue Liu:** data curation, formal analysis, writing – review and editing, conceptualization, methodology, investigation, validation, visualization, project administration. **Yun‐Shan Zhong:** writing – review and editing, investigation, validation, formal analysis, data curation. **Ming‐Ze Li:** formal analysis, data curation. **Jia‐Jun Lai:** formal analysis, data curation. **Yan‐Yu Luo:** formal analysis, data curation. **Shao‐Li Huang:** formal analysis, data curation. **Shao‐Qing Liu:** data curation, formal analysis. **Guo‐Hui Yu:** funding acquisition, supervision, resources, data curation, writing – review and editing, visualization, project administration, formal analysis, conceptualization, investigation, methodology. **Yun‐Hao Sun:** formal analysis, data curation. **Ming‐Wei Shao:** conceptualization, investigation, writing – review and editing, visualization, project administration, methodology, formal analysis, supervision, resources, data curation, writing – original draft, validation.

## Ethics Statement

The authors have nothing to report.

## Conflicts of Interest

The authors declare no conflicts of interest.

## Supporting information


**Figure S1:** Surfactin biosynthesis and assembly process; (A): Three kinds of surfactin of *B. subtilis*; (B): Surfactin biosynthesis and assembly process.
**Figure S2:** Results of surface activator hyperproduction vector construction; (A): Hyperproduction vector construction, (1): pHT315‐comK vector assay, positive result: 1349 bp; (2): pHT315‐phrC vector assay, positive result: 893 bp; (3): pHT315‐*comKphrC* vector assay, positive result: 1512 bp; (B): (1) Purpose of the three hyperproduction vectors band insertion and positive transformants, with positive results of 1349 bp, 893 bp and 1512 bp respectively; (2) is the detection of *B. subtilis* R31, with a positive score: 1382 bp; (C): Western blot detection of the expression of the target genes, a is the result of the detection of the target proteins, from left to right, Mock1 and Mock2 represent the R31 WT strains, and the R31 (pHT315) strain as blank control, *comK* protein expression in R31 (pHT315‐comK) strain, *phrC* protein expression in R31 (pHT315‐phrC) strain and *comK* and *phrC* protein expression in R31 (pHT315‐*comKphrC*) strain; b is the result of reference protein detection; (D): qRNA detection Results, from left to right, Mock1 and Mock2 represent the R31 WT strain, and the R31 (pHT315) strain is the blank control: The error line in the graphs is the mean ± standard deviation, *n* = 3; capital letters represent the significance level of significance of *p* < 0.05.
**Figure S3:** Principal component analysis (PCA) was performed using R based on gene expression information. The information of tens of thousands of dimensions (expression levels of tens of thousands of genes) contained in the sample is reduced to the comprehensive indicators of several dimensions (principal components), so as to conduct comparison between samples, and analyse the repeatability between repeated samples within the group and the difference between samples within the group. The first principal component (PC1); Ordinate: Second principal component (PC2), PC1 and PC2 could explain 88% of the total variance, judging that the sample had good repeatability, no outlier samples and significant differences between groups, *n* = 3.
**Figure S4:** KEGG database was used for functional annotation and classification of differentially expressed genes in different R31 overexpression strains. To study the distribution of differentially expressed genes in metabolic pathways and signal transduction pathways. After correction for multiple testing, the pathway with Qvalue ≤ 0.05 was selected as the threshold, and 23 pathways were defined as significantly enriched in the differentially expressed transcripts, *n* = 3.
**Figure S5:** For the assembly and clustering of transcriptome quality control in the control group and the R31 treatment group. To ensure the reliability of the analysis results, raw data (raw data) needs to be quality controlled before information analysis. The quality control software fastp (https://github.com/OpenGene/fastp) is used to reduce data noise and obtain clean data for subsequent information analysis. Reads containing adapters are removed, and the adapter and the subsequent part are truncated. 2. If the length of the truncated reads is less than 50, discard the reads; otherwise, retain them; remove all reads with only A bases; remove reads with a proportion of N greater than 10%; remove low‐quality reads (the number of bases with a quality value Q ≤ 20 accounting for more than 50% of the entire reads). Valid labels. After obtaining OTUs, perform OTU capacity statistics based on valid labels. Read QC filtering, low‐quality reads; nonoverlapping, unassembled reads; QC filtering of labels, labels that have not passed ‘label filtering’; false consensus labels, the number of false consensus labels; valid labels, the number of labels of valid data.
**Figure S6:** The transcriptome gene expression levels of the control group and the R31 treatment group were statistically analysed. The calculation of gene expression levels used the FPKM (Fragments Per Kilobase of transcript per Million mapped reads) method. Let FPKM (A) represent the expression level of gene A, then C is the number of fragments mapped to gene A, N is the total number of fragments mapped to known genes and L is the base number of gene A. The FPKM method can eliminate the influence of differences in gene length and sequencing quantity on the calculation of gene expression, and the calculated gene expression levels can be directly used to compare the differences in gene expression among different samples.
**Figure S7:** Statistical analysis was conducted on the differences in gene expression levels between the control group and the R31 treatment group. The edgeR software was used to perform differential analysis of gene expression levels between the groups. The differentially expressed genes were screened using FDR and log2FC. The screening conditions were FDR < 0.05 and |log2FC| > 1.
**Figure S8:** Splicing and clustering of quality control of bacterial communities in control and R31‐treated groups. The abscissa of the stack plot is the sample classification name, the ordinate represents the percentage and value and the different colours represent the data preprocessing classification. After raw reads were obtained by sequencing, we first filtered the low‐quality reads, then assembled them, spliced the double‐end reads into tags and then filtered the tags. The obtained data were called clean tag. Clustering was performed based on clean tag to remove the chimera tag detected during the clustering process, and the obtained data were Effective tag. After OTU was obtained, OTU abundance statistics were performed based on effective tag. Reads QC filter, low quality reads; Nonoverlap, unassembled reads without overlap; Tag QC filter, tags that do not pass ‘tag filter’; Chimera, tag number of chimera; Effective tag, the number of tags of effective data.
**Figure S9:** Rarefaction curve of Alpha diversity. Alpha diversity measured by the richness Chao1, Shannon and PD‐tree. A certain amount of sequencing data is randomly extracted from samples, and their alpha diversity index values are counted. The horizontal axis represents the amount of sequencing data and the vertical axis represents the corresponding alpha diversity index. When the curve flattens or reaches a plateau, the sequencing depth can be considered to have basically covered all species in the sample.

**Table S1:** Data preprocessing, statistics and quality control in the transcriptome sample.
**Table S2:** Tags details table in the transcriptome sample.
**Table S3:** Construct sequence of the vector.
**Table S4:** Inhibition rates of *B. subtilis* R31 wild‐type and surfactin overexpressed strains against *F. oxysporum* XJZ2.
**Table S5:** Biocontrol statistics of *B. subtilis* R31 wild‐type and surfactin overexpressed strains against *F. oxysporum* XJZ2.
**Table S6:** Data preprocessing, statistics and quality control in the microbiome sample.
**Table S7:** Tags details table in the microbiome sample.
**Table S8:** Alpha diversity index of banana soil in each treatment.
**Table S9:** Alpha diversity index of banana root in each treatment (R).
**Table S10:** Alpha diversity index of banana rhizosphere soil in each treatment (RS).

## Data Availability

The data that support the findings of this study are openly available in microbiomics data at https://www.ncbi.nlm.nih.gov/bioproject/1062623, reference number PRJNA126745.
