# Supplementary material for: Modulating Surfactin Biosynthesis in Bacillus subtilis R31 Enhances Behavioural Traits and Biocontrol Efficacy Against Banana Fusarium Wilt
Source: Microb Biotechnol. 2025 Nov 6;18(11):e70261. doi: 10.1111/1751-7915.70261 (PMC12592238; doi:10.1111/1751-7915.70261)
Supplement: Supplementary file 1 — Figure S1: Surfactin biosynthesis and assembly process; (A): Three kinds of surfactin of B. subtilis ; (B): Surfactin biosynthesis and assembly process. Figure S2: Results of surface activator hyperproduction vector construction; (A): Hyperproduction vector construction, (1): pHT315‐comK vector assay, positive result: 1349 bp; (2): pHT315‐phrC vector assay, positive result: 893 bp; (3): pHT315‐comKphrC vector assay, positive result: 1512 bp; (B): (1) Purpose of the three hyperproduction vectors band insertion and positive transformants, with positive results of 1349 bp, 893 bp and 1512 bp respectively; (2) is the detection of B. subtilis R31, with a positive score: 1382 bp; (C): Western blot detection of the expression of the target genes, a is the result of the detection of the target proteins, from left to right, Mock1 and Mock2 represent the R31 WT strains, and the R31 (pHT315) strain as blank control, comK protein expression in R31 (pHT315‐comK) strain, phrC protein expression in R31 (pHT315‐phrC) strain and comK and phrC protein expression in R31 (pHT315‐comKphrC) strain; b is the result of reference protein detection; (D): qRNA detection Results, from left to right, Mock1 and Mock2 represent the R31 WT strain, and the R31 (pHT315) strain is the blank control: The error line in the graphs is the mean ± standard deviation, n = 3; capital letters represent the significance level of significance of p < 0.05. Figure S3: Principal component analysis (PCA) was performed using R based on gene expression information. The information of tens of thousands of dimensions (expression levels of tens of thousands of genes) contained in the sample is reduced to the comprehensive indicators of several dimensions (principal components), so as to conduct comparison between samples, and analyse the repeatability between repeated samples within the group and the difference between samples within the group. The first principal component (PC1); Ordinate: Second principal component (P [file MBT2-18-e70261-s002.docx]

**Supplementary Information to**

**Modulating surfactin biosynthesis in *Bacillus subtilis* R31 enhances behavioral traits and biocontrol efficacy against banana *Fusarium* wilt**

Hao-Jun Chen^1^，Liu Yue^1^，Yun-Shan Zhong^1^, Ming-Ze Li^1^, Jia-Jun Lai^1^, Yan-Yu Luo^2^, Shao-Li Huang^2^, Shao-Qing Liu^2^, Guo-Hui Yu^1^, Yun-Hao Sun^1^, Ming-Wei Shao^1^*

^1^College of Agriculture and Biology, Key Laboratory of Green Prevention and Control on Fruits and Vegetables in South China, Ministry of Agriculture and Rural Affairs, Zhongkai University of Agriculture and Engineering, Guangzhou, China

^2^Guangzhou Academy of Agricultural and Rural Sciences, Guangzhou, China

**Correspondence**

Ming-Wei Shao

No. 24, Dongsha Street Guangzhou 510225, China

Email address: [jianting880720@126.com](mailto:jianting880720@126.com)


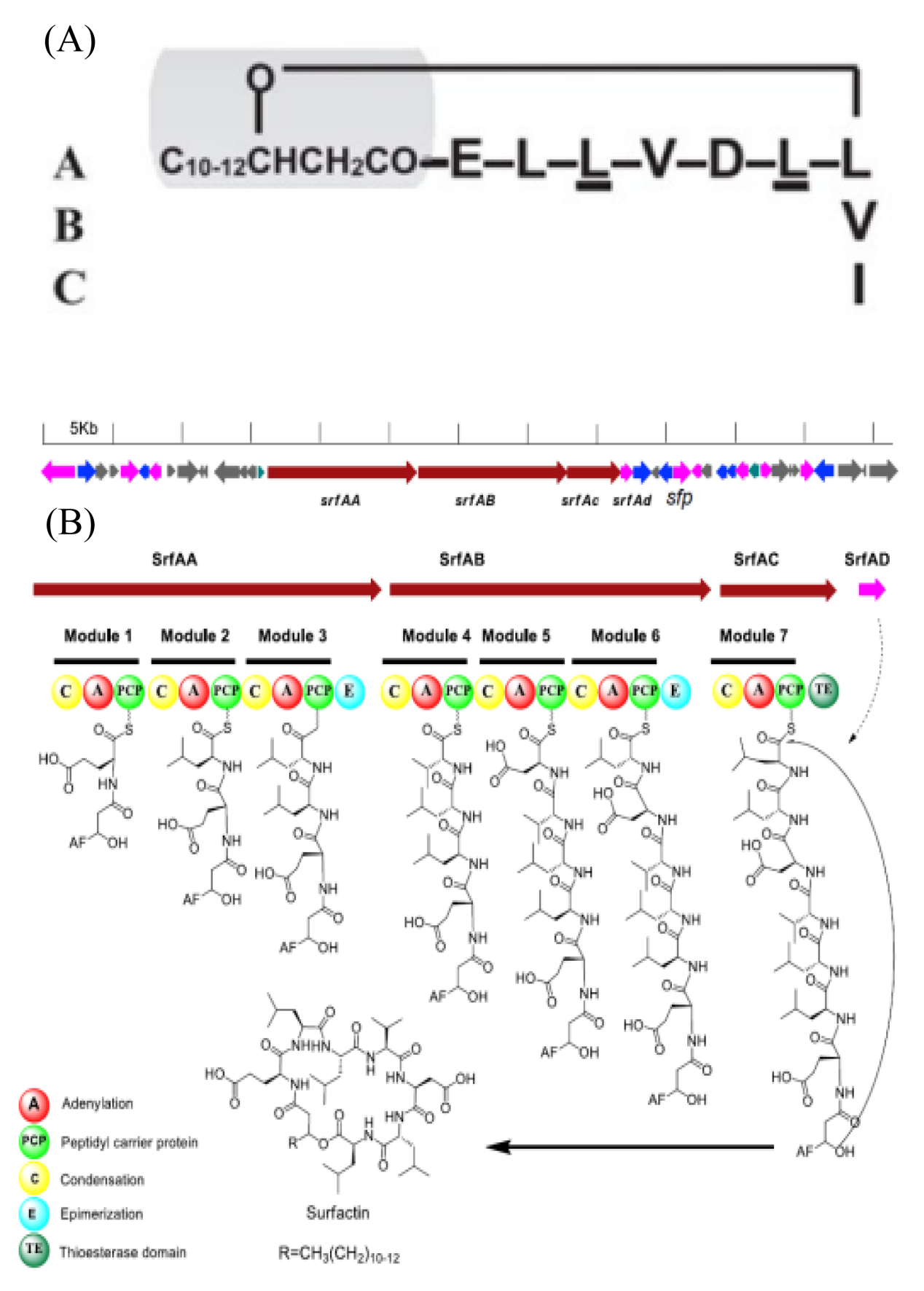


**Supplementary Figure 1** surfactin biosynthesis and assembly process; (A): Three kinds of surfactin of *B. subtilis;* (B): surfactin biosynthesis and assembly process


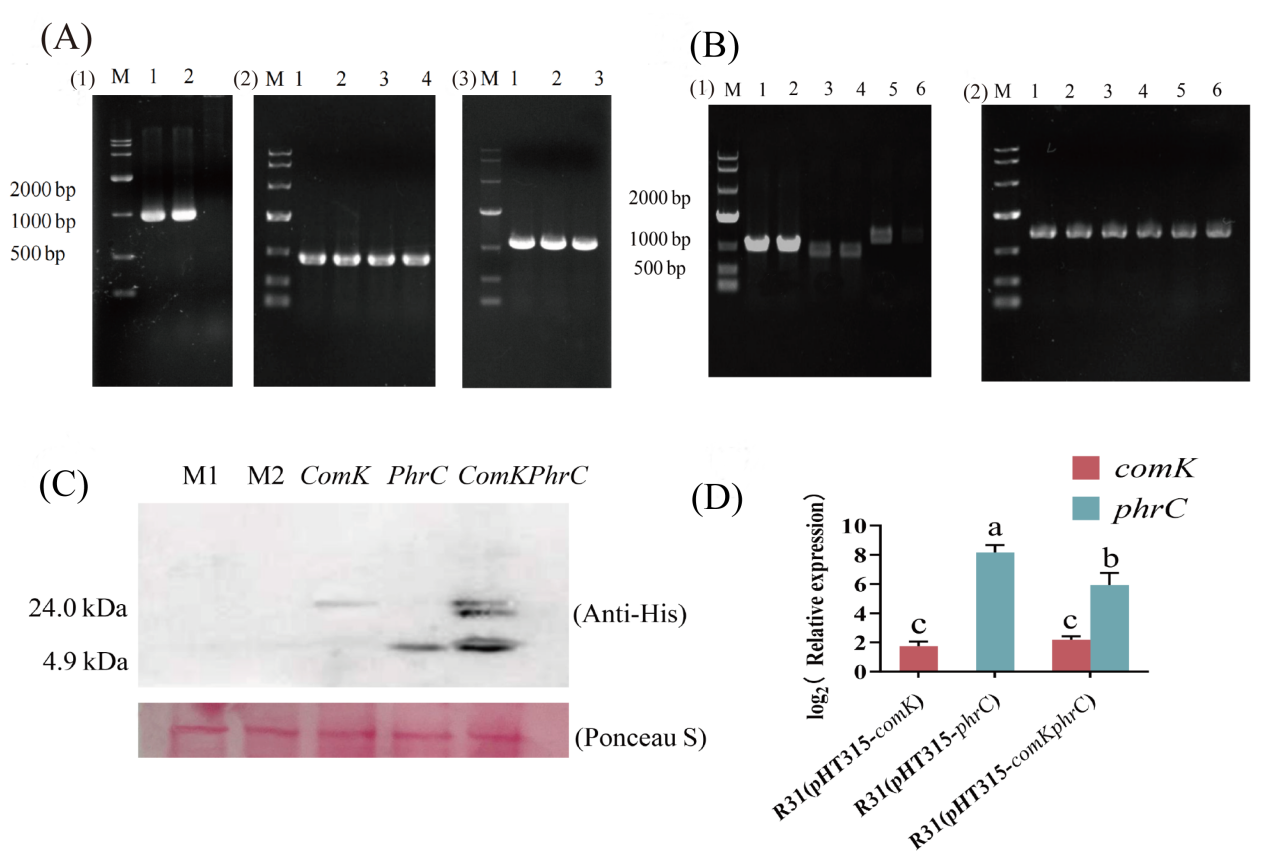


**Supplementary Figure 2** Results of surface activator hyperproduction vector construction; (A): hyperproduction vector construction, (1): pHT315-comK vector assay, positive result: 1349 bp; (2): pHT315-phrC vector assay, positive result: 893 bp; (3): pHT315-comKphrC vector assay, positive result: 1512 bp; (B) : (1) Purpose of the three hyperproduction vectors band insertion and positive transformants, with positive results of 1349 bp, 893 bp and 1512 bp, respectively; (2) is the detection of B. subtilis R31, with a positive score: 1382 bp; (C): Western blot detection of the expression of the target genes, a is the result of the detection of the target proteins, from left to right, Mock1 and Mock2 represent the R31 WT strains, and the R31 ( pHT315) strain as blank control, comK protein expression in R31 (pHT315-comK) strain, phrC protein expression in R31 (pHT315-phrC) strain, and comK and phrC protein expression in R31 (pHT315-comKphrC) strain; b is the result of reference protein detection; (D): qRNA detection Results, from left to right, Mock1 and Mock2 represent the R31 WT strain, and the R31 (pHT315) strain is the blank control: the error line in the graphs is the mean ± standard deviation, n=3; capital letters represent the significance level of significance of *P* < 0. 05.


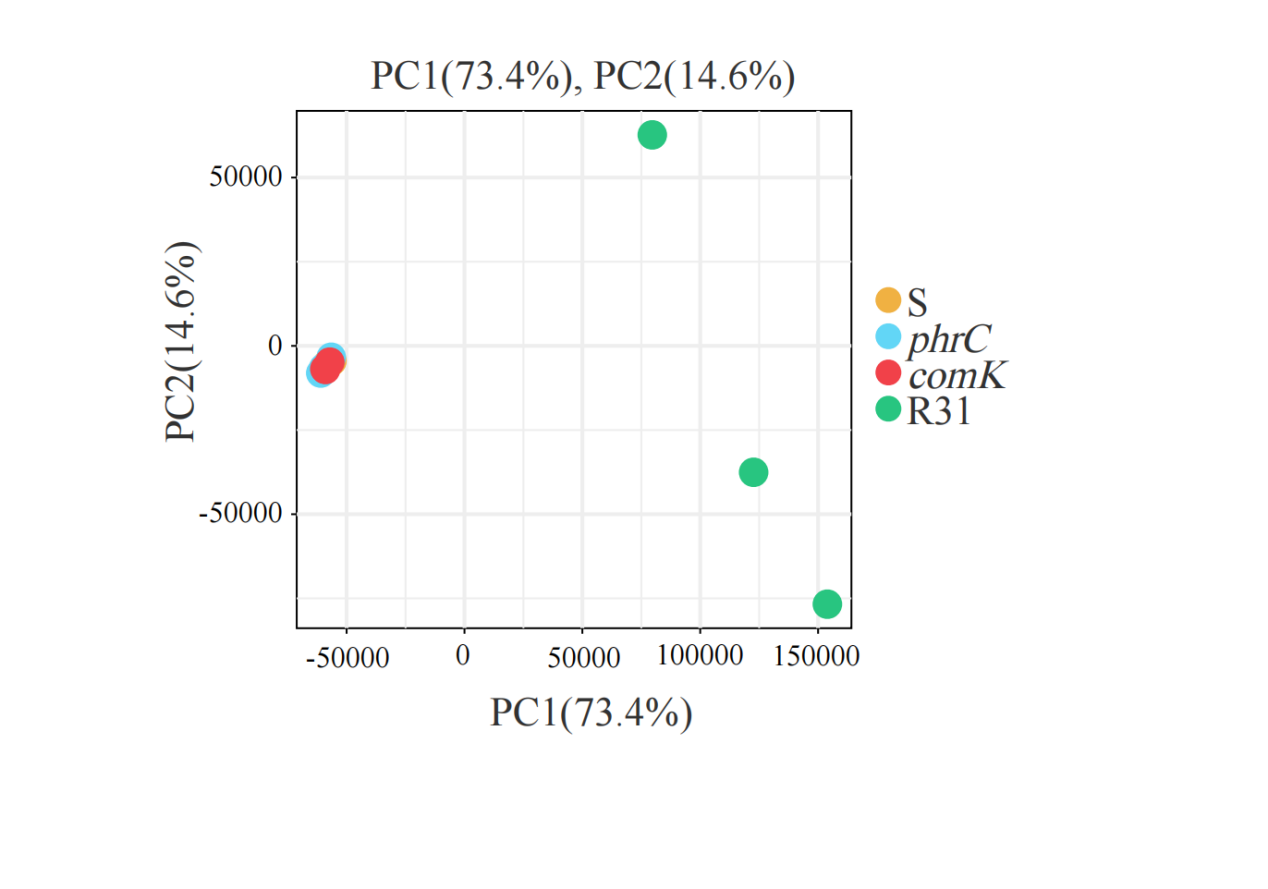


**Supplementary Figure 3**: Principal Component Analysis (PCA) was performed using R based on gene expression information. The information of tens of thousands of dimensions (expression levels of tens of thousands of genes) contained in the sample is reduced to the comprehensive indicators of several dimensions (principal components), so as to conduct comparison between samples, and analyze the repeatability between repeated samples within the group and the difference between samples within the group.The first principal component (PC1); Ordinate: Second principal component (PC2), PC1 and PC2 could explain 88% of the total variance, judging that the sample had good repeatability, no outlier samples, and significant differences between groups, n=3;


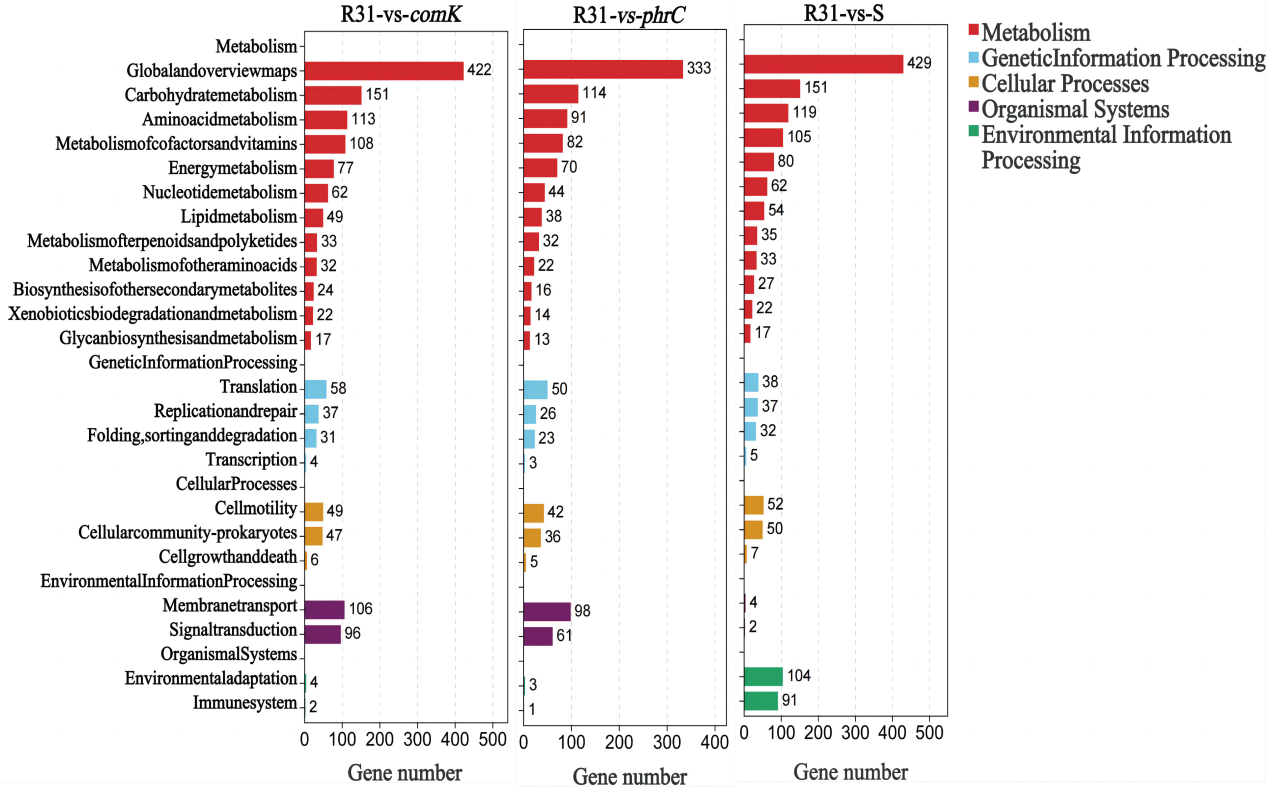


**Supplementary Figure 4.** KEGG database was used for functional annotation and classification of differentially expressed genes in different R31 overexpression strains.To study the distribution of differentially expressed genes in metabolic pathways and signal transduction pathways. After correction for multiple testing, the Pathway with Qvalue≤0.05 was selected as the threshold, and 23 pathways were defined as significantly enriched in the differentially expressed transcripts, n=3.


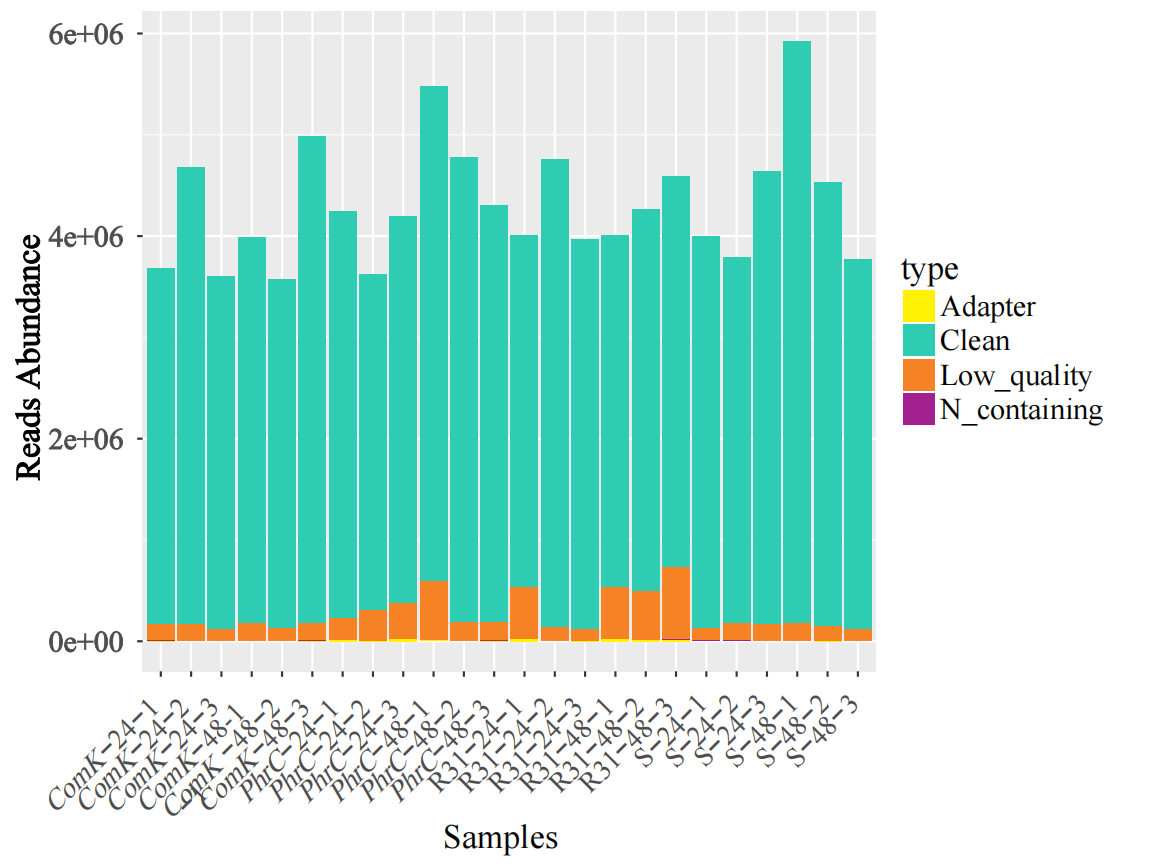


**Supplementary Figure 5**: For the assembly and clustering of transcriptome quality control in the control group and the R31 treatment group. To ensure the reliability of the analysis results, raw data (raw data) needs to be quality controlled before information analysis. The quality control software fastp (https://github.com/OpenGene/fastp) is used to reduce data noise and obtain clean data for subsequent information analysis. Reads containing adapters are removed, and the adapter and the subsequent part are truncated. 2. If the length of the truncated reads is less than 50, discard the reads; otherwise, retain them; remove all reads with only A bases; remove reads with a proportion of N greater than 10%; remove low-quality reads (the number of bases with a quality value Q ≤ 20 accounting for more than 50% of the entire reads). Valid labels. After obtaining OTUs, perform OTU capacity statistics based on valid labels. Read QC filtering, low-quality reads; non-overlapping, unassembled reads; QC filtering of labels, labels that have not passed "label filtering"; false consensus labels, the number of false consensus labels; valid labels, the number of labels of valid data.


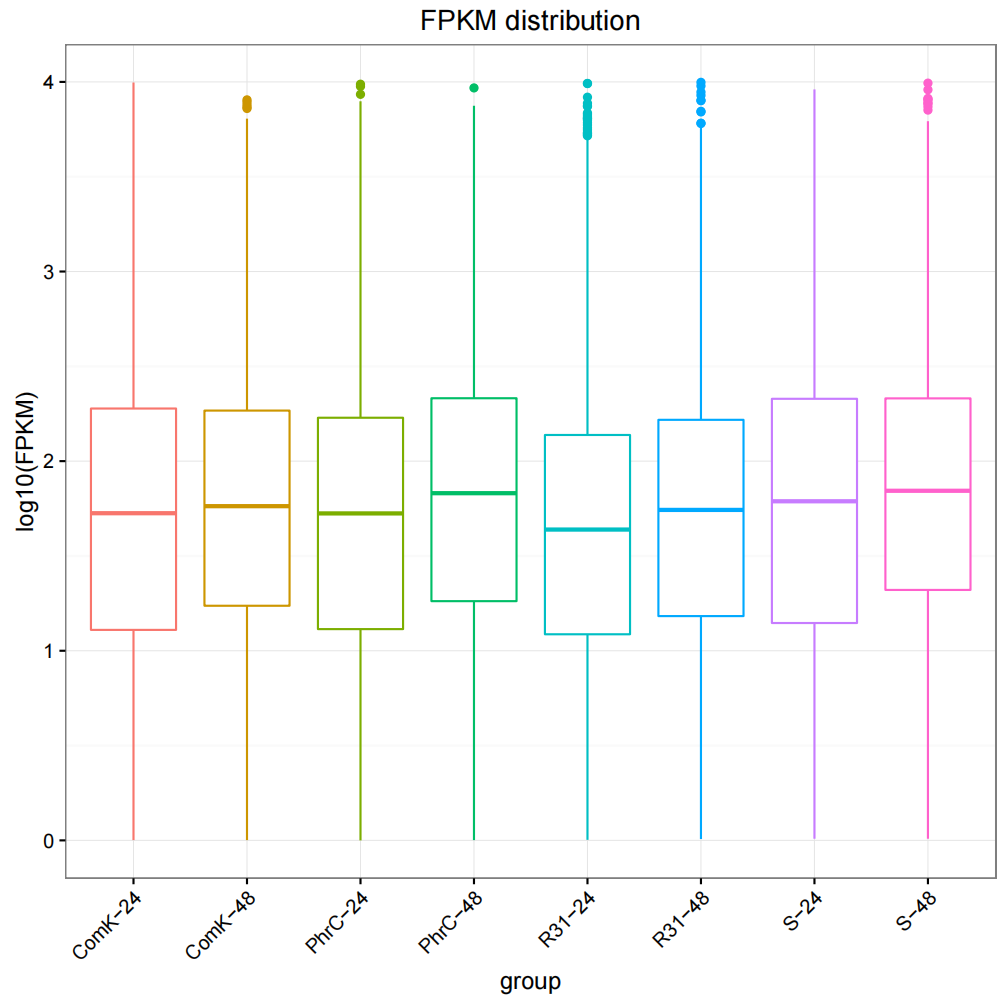


**Supplementary Figure 6**: The transcriptome gene expression levels of the control group and the R31 treatment group were statistically analyzed. The calculation of gene expression levels used the FPKM (Fragments Per Kilobase of transcript per Million mapped reads) method. Let FPKM(A) represent the expression level of gene A, then C is the number of fragments mapped to gene A, N is the total number of fragments mapped to known genes, and L is the base number of gene A. The FPKM method can eliminate the influence of differences in gene length and sequencing quantity on the calculation of gene expression, and the calculated gene expression levels can be directly used to compare the differences in gene expression among different samples.


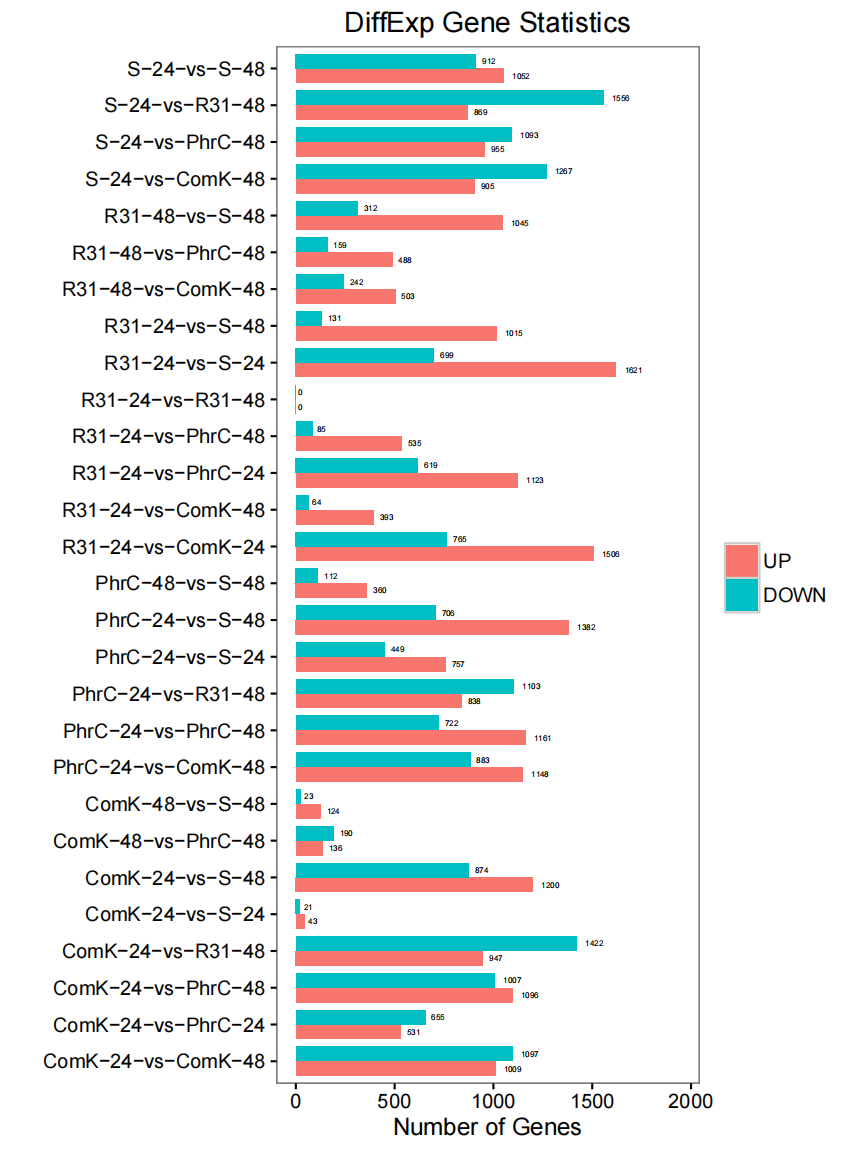


**Supplementary Figure 7**: Statistical analysis was conducted on the differences in gene expression levels between the control group and the R31 treatment group. The edgeR software was used to perform differential analysis of gene expression levels between the groups. The differentially expressed genes were screened using FDR and log2FC. The screening conditions were FDR < 0.05 and |log2FC| > 1.


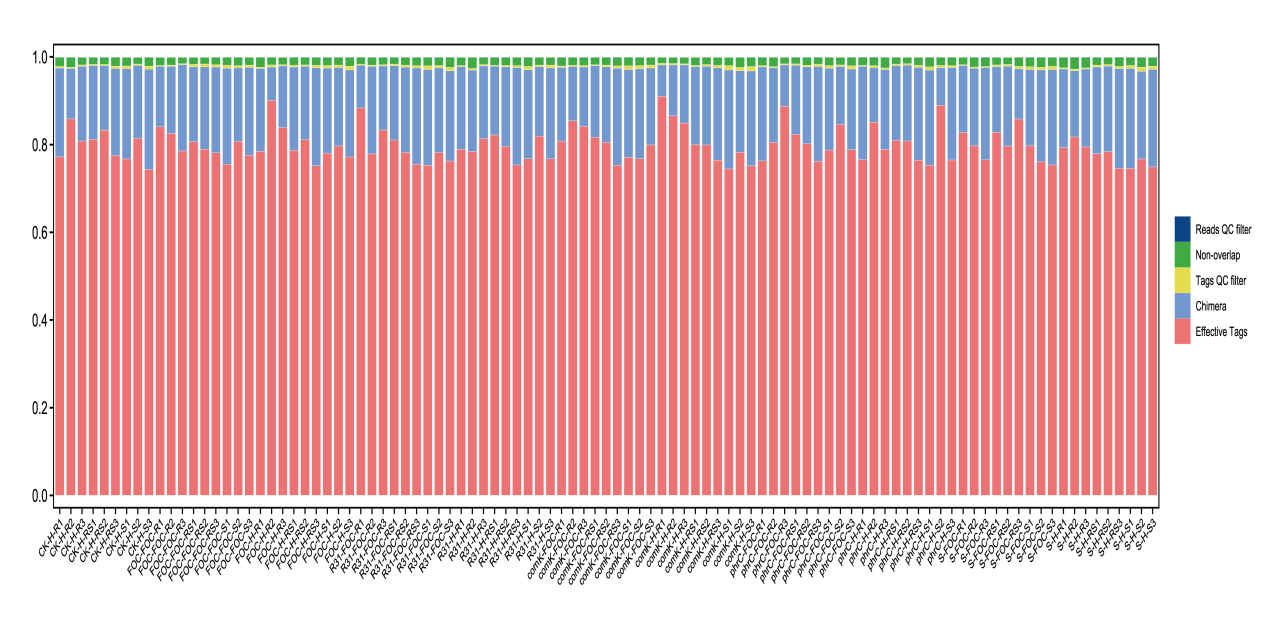


**Supplementary Figure 8**: Splicing and clustering of quality control of bacterial communities in control and R31-treated groups. The abscissa of the stack plot is the sample classification name, the ordinate represents the percentage and value, and the different colors represent the data preprocessing classification. After raw reads were obtained by sequencing, we first filtered the low-quality reads, then assembled them, spliced the double-end reads into tags, and then filtered the tags. The obtained data was called clean tag. Clustering was performed based on clean tag to remove the chimera tag detected during the clustering process, and the obtained data was Effective tag. After OTU was obtained, OTU abundance statistics were performed based on Effective tag. Reads QC filter, low quality reads; Non-overlap, unassembled reads without overlap; Tag QC filter, tags that do not pass “tag filter”; Chimera, tag number of chimera; Effective tag, the number of tags of effective data.


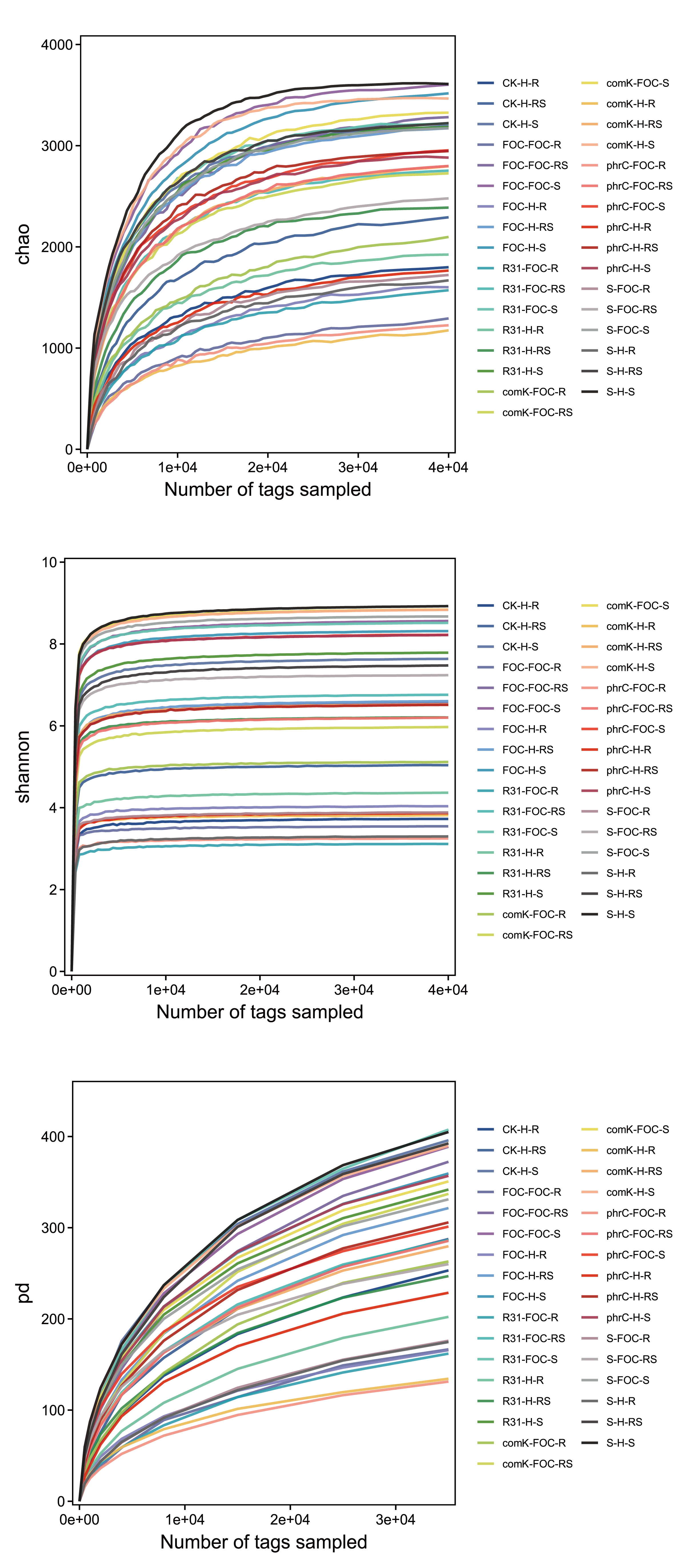


**Supplementary Figure 9**: Rarefaction curve of Alpha diversity. Alpha diversity measured by the richness Chao1, Shannon and PD-tree. A certain amount of sequencing data is randomly extracted from samples, and their alpha diversity index values are counted. The horizontal axis represents the amount of sequencing data and the vertical axis represents the corresponding alpha diversity index. When the curve flattens or reaches a plateau, the sequencing depth can be considered to have basically covered all species in the sample.
